# Abscisic acid pathway involved in the regulation of watermelon fruit ripening and quality trait evolution

**DOI:** 10.1371/journal.pone.0179944

**Published:** 2017-06-29

**Authors:** Yanping Wang, Shaogui Guo, Shouwei Tian, Jie Zhang, Yi Ren, Honghe Sun, Guoyi Gong, Haiying Zhang, Yong Xu

**Affiliations:** National Engineering Research Center for Vegetables, Beijing Academy of Agriculture and Forestry Sciences, Key Laboratory of Biology and Genetic Improvement of Horticultural Crops (North China), Beijing Key Laboratory of Vegetable Germplasm Improvement, Beijing, China; Zhejiang University, CHINA

## Abstract

Watermelon (*Citrullus lanatus* (Thunb.) Matsum. & Nakai) is a non-climacteric fruit. The modern sweet-dessert watermelon is the result of years of cultivation and selection for fruits with desirable qualities. To date, the mechanisms of watermelon fruit ripening, and the role of abscisic acid (ABA) in this process, has not been well understood. We quantified levels of free and conjugated ABA contents in the fruits of cultivated watermelon (97103; *C*. *lanatus* subsp. *vulgaris*), semi-wild germplasm (PI179878; *C*. *lanatus* subsp. *mucosospermus*), and wild germplasm (PI296341-FR; *C*. *lanatus* subsp. *lanatus*). Results showed that ABA content in the fruits of 97103 and PI179878 increased during fruit development and ripening, but maintained a low steady state in the center flesh of PI296341-FR fruits. ABA levels in fruits were highest in 97103 and lowest in PI296341-FR, but no obvious differences in ABA levels were observed in seeds of these lines. Examination of 31 representative watermelon accessions, including different *C*. *lanatus* subspecies and ancestral species, showed a correlation between soluble solids content (SSC) and ABA levels in ripening fruits. Furthermore, injection of exogenous ABA or nordihydroguaiaretic acid (NDGA) into 97103 fruits promoted or inhibited ripening, respectively. Transcriptomic analyses showed that the expression levels of several genes involved in ABA metabolism and signaling, including Cla009779 (*NCED*), Cla005404 (*NCED*), Cla020673 (*CYP707A*), Cla006655 (*UGT*) and Cla020180 (*SnRK2*), varied significantly in cultivated and wild watermelon center flesh. Three SNPs (-738, C/A; -1681, C/T; -1832, G/T) in the promoter region of Cla020673 (*CYP707A*) and one single SNP (-701, G/A) in the promoter of Cla020180 (*SnRK2*) exhibited a high level of correlation with SSC variation in the 100 tested accessions. Our results not only demonstrate for the first time that ABA is involved in the regulation of watermelon fruit ripening, but also provide insights into the evolutionary mechanisms of this phenomenon.

## Background

Due to its juicy, sweet and nutrient-rich flesh, watermelon (*Citrullus lanatus* (Thunb.) Matsum & Nakai) is among the most popular specialty fruits worldwide. However, the ancestral watermelon species (*C*. *lanatus* subsp. *lanatus*, also called as *C*. *lanatus* var. *citroides*, and *C*. *colocynthis* or other species) originated in Africa and fruits of these species are characterized by hard white flesh, thick rinds, low sugar content and a bitter flavor, all of which are important traits preventing the seeds from germinating and being damaged before the coming of the next rainy season [1]. Given the differences in fruit morphology and composition between the fruits of the ancestral species and the modern sweet-dessert watermelon, it is obvious that several significant genetic changes have occurred during the domestication of this specialty crop. The aggregate result of these changes is the conversion of the non-ripening, bitter wild watermelon into the fully ripening, sweet cultivated watermelon eaten by consumers today. Despite the importance of these genetic changes, relatively little is known about the evolutionary events leading to the generation of the modern watermelon. This lack of knowledge is due not only to a lack of information on the genes involved in watermelon ripening, but also due to a limited understanding of the physiology of watermelon fruit development.

The ripening of fleshy fruits is a complex and highly coordinated developmental process regulated at multiple levels (DNA, RNA, and protein), and dependent upon the coordinated activity of multiple plant hormones [2]. Over the past two decades, almost all of the “classical” phytohormones (i.e., abscisic acid [ABA], ethylene, auxin [IAA], cytokinins, and gibberellic acid [GA_3_]) have been reported to be either directly or indirectly involved in the regulation of fruit ripening in multiple species [3, 4]. Ethylene and ABA have been proven to play particularly important roles in the ripening of climacteric and non-climacteric fruits, respectively [3, 4]. In climacteric fruits, the onset of fruit ripening is accompanied by a dramatic increase in the respiratory rate of the fruit, coupled with the release of significant amounts of ethylene (referred to as “system II ethylene”) [3]. Treatments or mutations that impact either the production or the perception of system II ethylene can result in the inhibition of climacteric fruit ripening [3, 4]. In non-climacteric fruits, however, there is no respiratory burst during ripening, and the production of system II ethylene is not essential for fruit development. Instead, in several non-climacteric fruits, ABA has been demonstrated to play a critical role in regulating fruit ripening. In some non-climacteric fruits, such as grape, citrus, sweet cherry and cucumber, the levels of ABA present in developing fruits exhibits a dramatic increase (similar to the production of system II ethylene in climacteric fruits) during the onset of fruit ripening [5, 6, 7, 8]. This increase in ABA levels implies a potential role for this phytohormone in the regulation of fruit ripening. This hypothesis is supported by the fact that altering ABA levels in fruits through either chemical treatments or genetic manipulations (natural mutation, genetic engineering, *etc*.) results in alteration to the ripening process. In citrus, ABA-deficient mutant fruit displayed a slower rate of fruit degreening than did the wildtype; while treatments with exogenous ABA promoted fruit ripening by accelerating fruit coloration and significantly reducing organic acid content [9, 10]. Further, in grape, exogenous ABA application significantly promoted fruit ripening, and resulted in accelerated accumulation of both sugars and anthocyanins [11]. In cucumber, spraying turning stage fruits with ABA significantly reduced the level of chlorophyll present in the exocarp [8].

In addition to citrus fruits and grapes, studies in strawberries, another non-climacteric fruit, have provided particularly robust support for the importance of ABA in fruit ripening. For example, injecting strawberry fruits with either exogenous ABA or the ABA biosynthesis accelerator dimethyl sulphoxide (DMSO) notably accelerated fruit ripening; while injecting fruits with the ABA biosynthesis inhibitor nordihydroguaiaretic acid (NDGA) significantly suppressed fruit ripening [12, 13]. Moreover, down-regulation of *FaNCED1*, which encodes 9-*cis*-epoxycarotenoid dioxygenase (a rate-limiting enzyme in ABA biosynthesis), and *FaBG3*, which encodes β-glucosidase (an enzyme which hydrolyzes ABA glucose esters to release free ABA), led to the delay of fruit ripening and coloration, presumably due to a decrease of free ABA levels in these fruits [12, 14]. These data are supported by additional work performed in sweet cherry, where decreasing the transcriptional level of *PacCYP707A2*, which encodes a key enzyme in the oxidative catabolism of ABA, via VIGS (Virus Induced Gene Silencing), led to an increase in the ABA content and promoted the coloration and ripening of fruit [15]. Recent work has demonstrated that ABA is not only important in the ripening of non-climacteric fruits, but also plays an essential role in the ripening of several climacteric fruits, particularly tomato. In tomato fruits, changing the endogenous ABA level by either chemical treatments or genetic manipulation significantly affected several aspects of the ripening process, including the accumulation of carotenoids, the degradation of cell walls, and increase in the SSC (Soluble Solids Content) [16, 17].

Experimental evidence indicates that, in both climacteric and non-climacteric fruits, the effects of ABA on fruit ripening are dependent not only on ABA metabolism, but also on the presence of an intact ABA signaling pathway (i.e., perception and signal transduction). As a result, altering the expression levels of genes involved in ABA perception and/or signal transduction had been demonstrated to have a significant impact on fruit ripening. For example, in strawberry, VIGS-induced down-regulation of *FaCHLH*, a putative ABA binding magnesium chelatase H subunit in the CHLH-WRKY pathway, significantly inhibited the accumulation of anthocyanin and fruit ripening [12]. Additionally, decreased expression of a PYR/PYL/RCAR ABA receptor (*FaPYR*) and the protein phosphatase 2C/ABI1 ortholog *FaABI4* in strawberry resulted in decreased fruit ripening, a phenotype that was also observed following over expression of the SnRK2 kinase *FaSnRK2*.*6* [18–22].

Watermelon is a non-climacteric fruit [23, 24, 25], and the ripening mechanisms of this crop have not been well-studied. Based on the data presented above, we hypothesized that the accumulation of ABA in fruit tissues may play a critical role in the regulation of watermelon fruit ripening. This hypothesis was supported by the fact that ABA levels in watermelon have previously been found to peak at the onset of fruit ripening, coincident with dramatic increases in the expression levels of the *ClNCED4* and *ClCYP707A1* genes [26]. Watermelon provides an ideal system to study the effects of ABA on fruit ripening. While modern cultivated watermelon fruits can fully ripen, fruits of wild watermelon (primarily distributed in the northeast of Africa), do not ripen [27]. Thus, watermelon provides a very attractive system to investigate the physiological and genetic changes which occurred in watermelon during the domestication process, and which are responsible for watermelon fruit ripening.

In this study, we sought to reveal the role of ABA in regulating watermelon fruit ripening, as well as its potential role in the evolution of quality trait. We quantified ABA and ABA glucose esters (ABA-GE) levels in different fruit tissues that were collected from cultivated watermelons (97103; *C*. *lanatus* subsp. *vulgaris*), semi-wild germplasm (PI179878; *C*. *lanatus* subsp. *mucosospermus*), and wild germplasm (PI296341-FR; *C*. *lanatus* subsp. *lanatus*). Additionally, the effects of application of either exogenous ABA or NDGA on watermelon fruit ripening were evaluated. To elucidate how the fruit quality trait (mainly SSC) was impacted by ABA during the evolution process, the levels of ABA and SSC of ripening fruits from 31 representative watermelon accessions were quantified. At the molecular level, the expression levels of genes encoding key enzymes involving in the ABA metabolism were also investigated using real-time quantitative PCR (qPCR). Making use of watermelon transcriptomic libraries previously generated in our laboratory [27], we performed analyses to identify specific transcripts and genes related the role of ABA in fruit ripening as well as in the evolution of quality trait. The results presented here are not only useful in clarifying the mechanisms of watermelon fruit ripening regulation, but also expand our understating of the evolution and domestication of modern watermelon.

## Materials and methods

### Plant materials

Watermelon plants, *C*.*lanatus* (Thunb.) Matsum.&Nakai subsp. *vulgaris* cv 97103, *C*. *lanatus* subsp. *mucosospermus* semi-wild germplasm PI179878, and *C*. *lanatus* subsp. *lanatus* (also referred to as *C*. *lanatus* var. *citroides*) wild germplasm PI296341-FR, were grown in plastic greenhouse containing a standard potting mix (peat:sand:pumice, 1:1:1, v/v/v). Watermelon line 97103, a typical normal-ripening cultivar, has four critical fruit development and ripening stages delineated by changes in color, texture, flavor, aroma, and other fruit ripening characteristics (described in detail by Guo et al. [27]): 1. immature white (10 days after pollination, DAP); 2. white-pink flesh (18 DAP); 3. red flesh (26 DAP); and 4. ripe (34 DAP). However, watermelon line PI296341-FR is a wild type variety, which exhibits a non-ripening phenotype [27]. Watermelon line PI179878 is defined as semi-wild, and exhibits intermediate ripening phenotype, more similar to the cultivated watermelon, but not fully ripening. Flowers were hand-pollinated and tagged on the day of pollination. Fruits of uniform size and without mechanical damage were periodically harvested (1, 3, 5, 10, 18, 26, 34, 42 and 50 DAP), and at least three fruits were collected for each developmental stage. The center flesh, mesocarp and placenta were separated from fruits immediately after harvest (fruits of 1, 3 and 5 DAP were hard to separate and different fruit tissues were collected as a whole), frozen in liquid nitrogen and stored at -80°C for further use.

Fruits of 31 watermelon accessions representing three different *C*. *lanatus* subspecies and ancestral species were harvested at the ripening stage, and center flesh was immediately separated, frozen in liquid nitrogen and stored at -80°C for further use. At least three fruits were collected for each accession. Accession information are listed in S1 Table.

### Exogenous ABA and NDGA treatments

Watermelon fruits of 97103 grown under standard greenhouse conditions were treated at 15 DAP by injecting: a. 1 mL 400 mg/L ABA (Part # A1049, Sigma-Aldrich, St. Louis, MO; ABA treatment), b. 1 mL 100 mg/L NDGA (Part # 74540, Sigma-Aldricih, St. Louis, MO; ABA synthesis inhibitor treatment); or sterile water (control treatment). At least 10 fruits were treated and tagged for each treatment. Samples were collected three days after treatment.

### Determination of the SSC and firmness of the center flesh

The SSC of center flesh tissues was determined using a hand-held ATC-1E refractometer (ATAGO, Tokyo), according to the manufacturer’s instructions. The center flesh firmness was determined using the FT-327 penetrometer (Bertuzzi, Facchini, Italy).

### Determination of ABA and ABA-GE content

Determination of ABA and ABA-GE content was conducted in the mass spectrometry (MS) laboratory of the College of Biological Sciences, China Agricultural University. Samples were harvested as described above, frozen in liquid nitrogen, and stored at -80°C. Samples were then ground into a fine powder in liquid nitrogen, and 100 mg of each sample was transferred into 1.5 mL microcentrifuge tube. 500 μL extraction solution 1 (isopropanol: H_2_O: HCl = 2: 1: 0.02, v/v/v) and 50 μL internal standard (100 μg/L) were added to each sample tube, and tubes were then sealed and vortexed with an oscillation of 10 s, vibration rate of 300 rpm for 30 min at 4°C. 1 mL extraction solution 2 (100% CHCl_3_) was then added to each tube, and tubes were then vortexed for an additional 30 min at 4°C (vortex oscillation 10 s, vibration at 300 rpm). Samples were then centrifuged at 14,000 rpm for 5 min, 4°C. Centrifugation led to the formation of two phases, and the lower, organic phase (approximately 1.2 mL) was harvested and transferred to a new microcentrifuge tube. Samples were then dried under nitrogen at room temperature, redissolved in 0.1 mL methanol, centrifuge for 5 min at 14,000 rpm and loaded into target vials. Determination of ABA-GE was achieved by adding an external standard method.

Chromatographic separation was performed on the Waters ACQUITY UPLC I-Class system (Waters Corporation, 34 Maple Street Milford, MA, 01757 USA) using a Poreshell EC-120 chromatographic column (3.0 x 100 mm, 3 μm; Agilent, Santa Clara, CA). Separation of ABA and ABA-GE was achieved using the following solvent gradient (solvent A: H_2_O contains 0.05% acetic acid, solvent B: acetonitrile contains 0.05% acetic acid): 0 minutes, 10% B; 6.25 minutes, 40% B; 7.50 minutes, 90% B; 10.5 minutes, 90% B; 10.6 minutes 10% B; 13.5 minutes, 10% B; with a flow rate of 0.3 mL/min and an injection volume of 5 μL. Mass spectrometric analyses were performed using a Thermo Q-Exactive high resolution mass spectrometer (Thermo Scientific, Waltham, MA, USA) and the following conditions: Ion Source, HESI; Spray Voltage (-), 3000; Capillary Temperature, 320; Sheath Gas, 30; Aux Gas, 10; Spare Gas, 5; Probe Heater Temp., 350; S-Lens RF Level, 55. The retention time and mass spectrometry information of ABA and ABA-GE were determined with standard substance ((±)-abscisic acid, A1049, Sigma, St Louis, MO, USA; ABA-GE, No. CDGP-0132781, OlChemIm). Each sample had three biological replicates.

### Total RNA extraction, RT-PCR and qRT-PCR analysis

Total RNA was extracted using the Huayueyang Quick RNA isolation Kit (Cat. No.: ZH120, Huayueyang Biotechnology, Beijing, China) following the manufacturer’s instructions. The quantity and quality of the total RNA were checked using a NanoDrop 1000 spectrophotometer (Thermo Fisher Scientific Inc.; USA) and by resolution on a 1% non-denaturing agarose gel, respectively. cDNA was synthesized from the total extracted RNA using the FastQuant RT Kit (with gDNase) (Tiangen Biotech, Beijing, China) according to the manufacturer’s instructions. qRT-PCR assays were performed using the LightCycler480 RT-PCR system (Roche, Switzerland) with specific primers (S2 Table). Each reaction consisted of 10 μL SYBR Green I Master Mix, 5 μL cDNA (20 ng/μL) and 5 μL primer mix (2 μM of each primer) to make a total volume of 20 μL. Reactions were carried out under the following conditions: 95°C for 5 min, followed by 40 cycles of 95°C for 20 sec, 60°C for 20 sec, and 72°C for 20 sec. PCR amplification of a single product of the correct size for *ClNCEDs* and *ClCYP707As* were confirmed by agarose gel electrophoresis and sequencing. Plasmids containing each specific gene were used in standard curve assays and transcripts of these genes were normalized as copy number per nanogram of total RNA (absolute quantification).

### Transcriptome sequencing data analysis

Transcriptomic libraries of the genes expressed in the center flesh of 97103 and PI296341-FR watermelon at different fruit developmental stages were previously generated by our group [27]. The expression profiles of ABA pathway genes were analyzed and visualized using MeV4.9.0 software [28].

### Analysis of the gene promoter sequences from wild, semi-wild and cultivated watermelon accessions

The promoter sequences of Cla009779 (*NCED*), Cla005404 (*NCED*), Cla020673 (*CYP707A*), Cla006655 (*UGT*) and Cla020180 (*SnRK2*) (approximately 2 kb per promoter sequence) were amplified from the genomic DNA of different watermelon accessions using Prime STAR HS DNA Polymerase (Takara). The resulting PCR products were then purified and sequenced. The quality of the sequencing reactions and data were then analyzed using the BioEdit Sequence Alignment Editor and low-quality sequences were removed. S2 Table lists the primers used for amplification. The promoter sequences were aligned using ClustalX.

## Results

### ABA content is well correlated with watermelon fruit development and ripening

**Fig 1:**
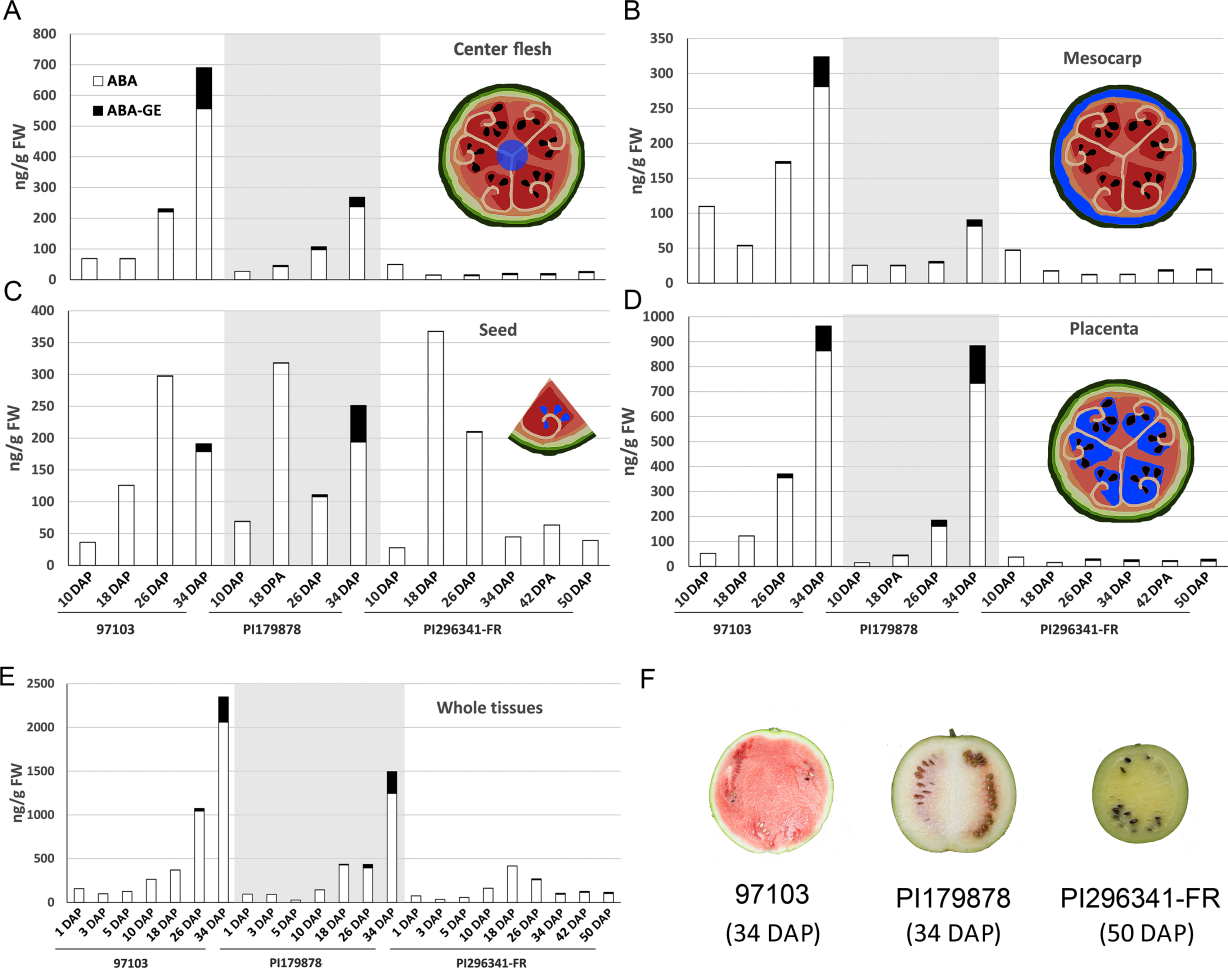
Accumulation of ABA and ABA-GE content in different types of watermelon fruits. ABA and ABA-GE content in center flesh (A), mesocarp (B), seed (C), placenta (D) and whole fruit (E) during 97103, PI179878 and PI296341-FR watermelon fruit development and ripening. (F) Pictures of 97103, PI179878 and PI296341-FR watermelon fruits at the ripening stage, respectively at 34, 34 and 50 DAP. Sampled tissues are indicated in blue.

To investigate the role of ABA in watermelon fruit development and ripening, levels of both free and conjugated ABA (ABA-GE) were quantified in different fruit tissues of 97103, PI179878 and PI296341-FR watermelons at distinct fruit development and ripening stages (1, 3, 5, 10, 18, 26, 34, 42 and 50 DAP). In all the tested samples, free ABA accounted for much of total ABA, while ABA-GE contents were relatively low (Fig 1). In the fleshy parts of the watermelon fruits, including the center flesh and placenta, the ABA contents in cultivated 97103 and semi-wild PI179878 increased throughout fruit development and ripening, reaching their highest levels at the ripening stage (34 DAP), while the ABA content in wild PI296341-FR was maintained at a relatively low steady state, with little variation (Fig 1A and 1D). In mesocarp tissues, the ABA content in 97103 was higher than in the other two species tested (Fig 1B). In seeds, the ABA content peaked at 26 DAP in 97103, while it was 8 days earlier in PI179878 and PI296341-FR and there was no distinct difference in absolute ABA content in seed in all species (Fig 1C). Overall, the whole fruit ABA content was the highest in 97103, followed by PI179878 and lowest in PI296341-FR (Fig 1E). These results suggested that variation in the ABA contents in fruits, particularly in the fleshy parts of the fruit, may be associated with watermelon fruit development and ripening.

### ABA content was positively correlated with SSC in wild, semi-wild and cultivated watermelon accessions

**Fig 2:**
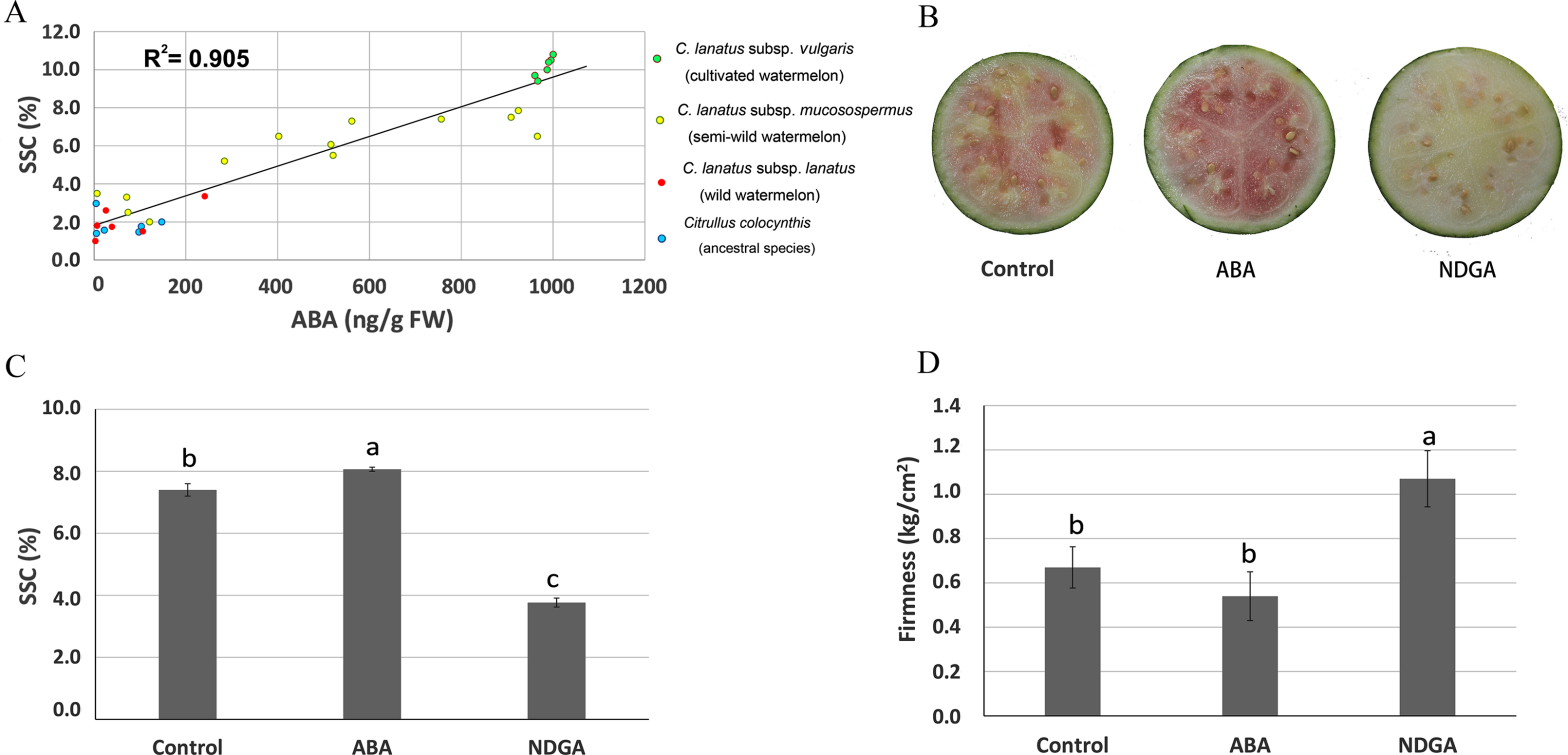
Effects of ABA on watermelon fruit ripening. (A) ABA and SSC content in six cultivated, thirteen semi-wild, six wild and six ancestral watermelon accessions at the ripening stage. Values are means of three independent replicates. Changes of fruit color (B), center flesh SSC (C) and firmness (D) in cultivated watermelon 97103 after ABA and NDGA treatment. Error bars on each point indicate ±SE from three independent replicates. Pairwise comparisons between the treatments were done with ANOVA and means were separated with Fisher’s test α< 0.05.

The evolution of watermelon fruits has resulted in major changes in fruit quality, particularly in sweetness of the ripened fruit [29, 30]. To further investigate the role of ABA in fruit ripening and formation of quality trait during watermelon evolution, we quantified the levels of both ABA and SSC in center flesh at the ripening stage in 31 representative watermelon accessions including cultivated, semi-wild, wild and ancestral watermelons. As is shown in Fig 2A, both ABA content and SSC were relatively low in wild and ancestral accessions, higher in cultivated accessions, and at intermediate levels in semi-wild accessions. Additionally, a rather good correlation (R^2^ = 0.905) was observed between ABA content and SSC in ripening watermelon fruits of different evolutionary stages. These results support the hypothesis that ABA may be involved in the regulation of watermelon fruit ripening and the evolution of main quality trait (SSC).

### ABA is involved in the regulation of cultivated watermelon fruit ripening

To further validate the role of ABA in regulating watermelon fruit ripening, either exogenous ABA or the ABA biosynthesis inhibitor NDGA were injected into 97103 fruits at 15 DAP. Fig 2B shows that exogenous ABA promoted fleshy part coloration, and that NDGA treatments inhibited this process. Additionally, exogenous ABA treatments significantly increased SSC in watermelon center flesh, whereas NDGA treatment notably reduced it (Fig 2C). Finally, the application of exogenous ABA slightly (but not significantly) reduced center flesh firmness, while NDGA treatments significantly increased firmness (Fig 2D). These results revealed that alterations in ABA content can affect cultivated watermelon fruit ripening.

### *NCED* and *CYP707A* genes are dynamically expressed in both mesocarp and fleshy tissues during watermelon fruit development and ripening

**Fig 3:**
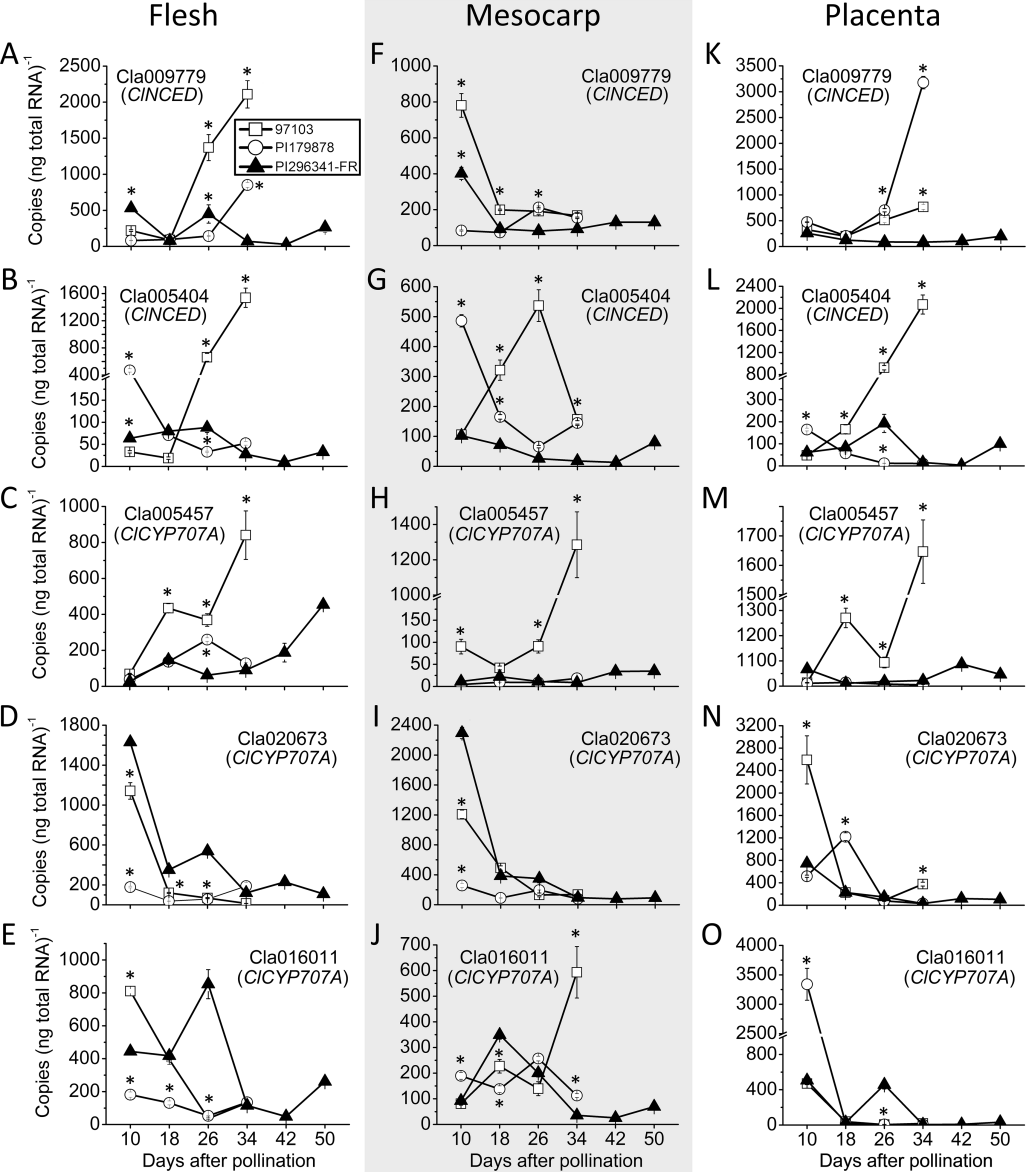
Expressions of key ABA metabolism genes in center flesh, mesocarp and placenta. Expression levels of Cla009779 (*ClNCED*), Cla005404 (*ClNCED*), Cla005457 (*ClCYP707A*), Cla020673 (*ClCYP707A*) and Cla016011 (*ClCYP707A*) in the center flesh (A, B, C, D, E), mesocarp (F, G, H, I, J) and placenta (K, L, M, N, O) of 97103, PI179878 and PI296341-FR during watermelon fruit development and ripening. Standard errors are indicated. * indicates expression values that are significantly different at the 0.01 level compared to PI196341-FR at each developmental stage.

To elucidate the molecular basis of the observed differences in ABA contents between 97103, PI179878 and PI296341-FR, we quantified the expression levels of *ClNCED* and *ClCYP707A* gene transcripts, which respectively encode the enzymes catalyzing the rate-limiting step in ABA biosynthesis pathway and the 8’-hydroxylation of ABA in the degradation pathway in these accessions. The *ClNCED* and *ClCYP707A* genes were identified from the watermelon genome database via BLAST search [31], utilizing the homologous genes of *Arabidopsis*, tomato, avocado and *Phaseolus vulgaris* as queries. Additionally, based on the RNA-Seq data previously generated in our laboratory [27], two additional *NCEDs* (Cla005404, Cla009779) and three additional *CYP707As* (Cla005457, Cla020673, Cla016011) with high expression levels were selected for further investigation via quantitative real-time PCR (Fig 3).

In center flesh, in the cultivated 97103, expression of the two *NCEDs* investigated (Cla009779 and Cla005404) exhibited a similar trend, increasing throughout fruit development, with peak expression occurring at 34 DAP (Fig 3A and 3B). In this same accession, the expression of the three *CYP707A* genes, Cla020673 and Cla016011 decreased during fruit ripening, with the highest value appearing at 10 DAP (Fig 3D and 3E); but expression of Cla005457 increased during fruit development and ripening and reached a maximum at 34 DAP (Fig 3C). In the semi-wild accession PI179878, the level of Cla009779 (*NCED*) expression was relatively low during the first three tested stages, but increased at 34 DAP (Fig 3A); while, the other *NCED* assayed, Cla005404, showed a decreasing pattern with the highest value at 10 DAP (Fig 3B). In this accession, the expression levels of *CYP707A* genes were, in general, lower than that of the cultivated 97103. However, the *CYP707A* genes exhibited varying patterns of expression, with Cla005457 increasing during fruit development and peaking at 26 DAP (Fig 3C), while both Cla020673 and Cla016011 had two peaks in expression, one at 10 DAP and another at 34 DAP (Fig 3D and 3E). In the wild accession PI296341, expression variance of the two *NCEDs* were relatively small (Fig 3A and 3B). In contrast, the expression levels of *CYP707As* dramatically changed during fruit development and ripening in this accession. Specially, Cla005457 increased during fruit ripening, and peaked at 34 DAP (Fig 3C); while Cla020673 exhibited a decreasing trend with the highest value occurring at 10 DAP (Fig 3D). Expression of Cla016011, peaked at 26 DAP, then decreased to the lowest level at 42 DAP, after which expression levels again increased (Fig 3E).

In placenta, expression levels of the *NCEDs* and *CYP707As* genes assayed were similar to those observed in center flesh across all accessions tested, with one exception. In the semi-wild accession, expression of the *CYP707A* (Cla020673) exhibited a zigzag expression pattern, with peaks at 18 and 34 DAP (Fig 3N).

In mesocarp, besides the two *CYP707As*, Cla005457 and Cla020673, most of the genes showed different expression patterns comparing to those in the center flesh and placenta in all the accessions tested. In 97103, expression of Cla009779 decreased from its highest level at 10 DAP to a lower level at 18 DAP, then it kept at a relatively stable level at other stages (Fig 3F), which was opposite from that in both center flesh and placenta. In this same accession, expression of Cla005404 increased from 10 DAP to 26 DAP, and then decreased to its lowest level at 34 DAP (Fig 3G). In the CYP707A family, Cla016011 showed an opposite trend comparing to that in the center flesh and placenta, and peaked at 34 DAP (Fig 3J). In the semi-wild PI179878, Cla005404 followed a similar pattern as in the center flesh (Fig 3G), while Cla009779 peaked at 26 DAP although the expression variation was relatively small (Fig 3F); in the CYP707A family, the Cla016011 showed a relatively stable expression pattern comparing to that in the center flesh and placenta (Fig 3J). In the wild accession, expression variance of both Cla009779 and Cla005404 were relatively small with peaks appearing at 10 DAP and 34 DAP respectively (Fig 3F and 3G). In regard to the Cla016011, it firstly increased from 10 DAP to 18 DAP, then decreased until 42 DAP, and after that it increased slightly at 50 DAP (Fig 3J).

Overall, from the perspective of absolute expression values of the five tested genes, no differences in magnitude was found, suggesting that those dynamically expressed genes may all involve in the regulation of ABA level during watermelon fruit ripening.

### Genes in the ABA metabolism and signal transduction pathways showed different expression profiles between cultivated and wild watermelons

In order to further explore the molecular mechanism by which ABA impacts fruit ripening and quality trait evolution (mainly refers to the changes of SSC from low to high during watermelon evolution), we identified several additional genes involved in the ABA metabolism and signal transduction from watermelon genome database [31], including genes encoding zeaxanthin epoxidase (ZEP), abscisic aldehyde oxidase (AO), MoCo sulfurase (MOSU), BG, glucosyltransferase (UGT), PYL, PP2C and SnRK2. Sequence alignments of the corresponding gene families are shown in S1–S10 Figs.

**Fig 4:**
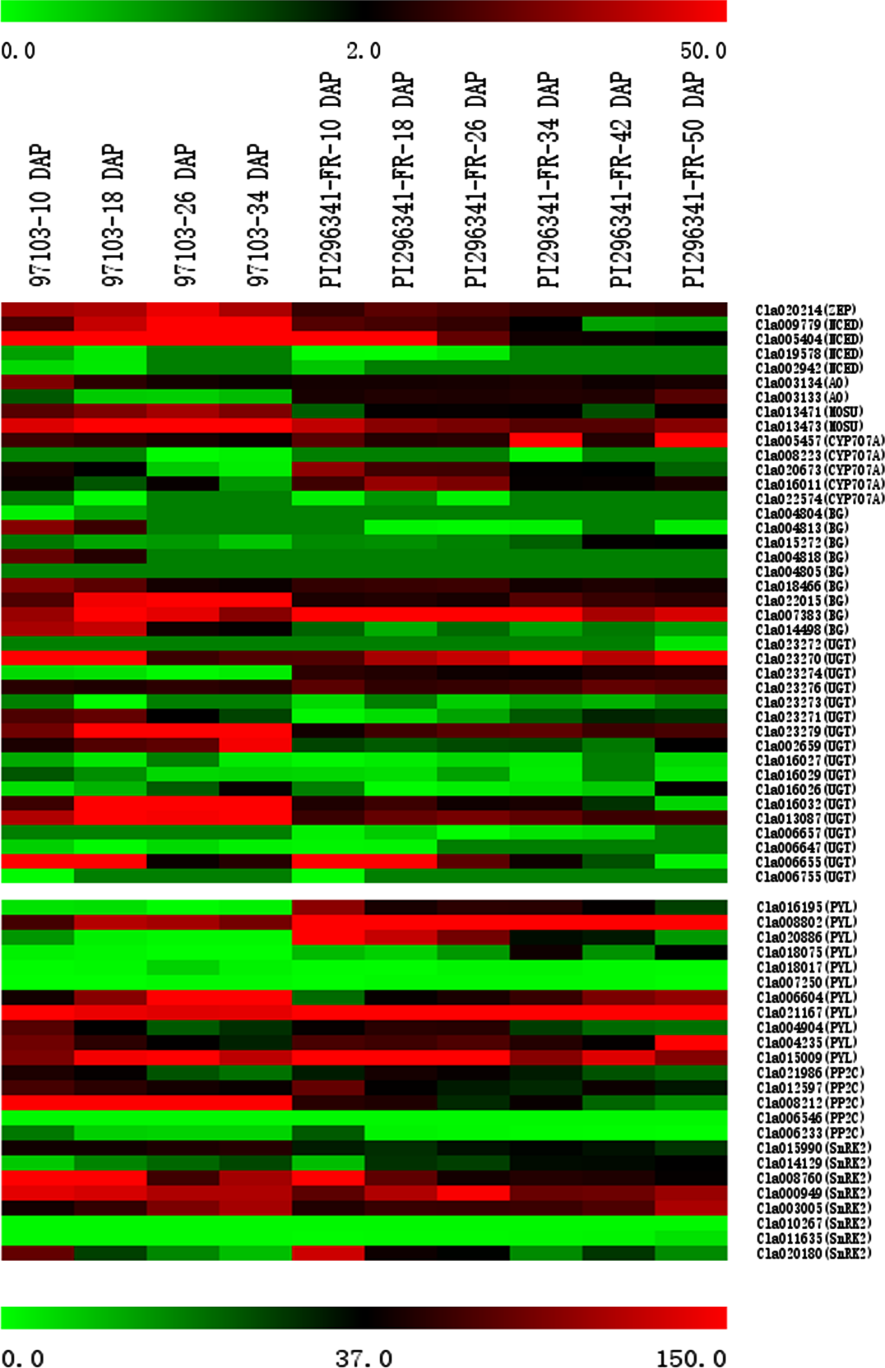
Expression patterns of ABA pathway genes. Expression profiles of key ABA metabolism and core ABA signal transduction genes across different watermelon developmental stages in 97103 and PI296341-FR center flesh. The scale representing the relative signal intensity values is shown above. DAP: Days After Pollination.

To gain insights into the expression profiles of ABA metabolism and signal transduction pathway genes in center flesh of 97103 and PI296341-FR during fruit development and ripening, we analyzed the RNA-seq data previously generated in our lab [27] (Fig 4). Most of the ABA pathway genes showed differential expression between 97103 and PI296341-FR in at least one developmental stage. Five genes, including four ABA metabolism genes and one signal transduction gene, were found to be not only dynamically expressed, but also differentially expressed in center flesh of 97103 and PI296341-FR during fruit development and ripening (27, Fig 4), namely Cla005404 (*NCED*), Cla009779 (*NCED*), Cla020673 (*CYP707A*), Cla006655 (*UGT*) and Cla020180 (*SnRK2*). The two *NCEDs* Cla005404 and Cla009779 exhibited similar expression patterns, and both were highly expressed and reached peak values before fruit ripening in 97103. However, both genes were expressed at relatively low levels, which gradually decreased in PI296341-FR. Expression of Cla020673 (*CYP707A*) was higher in PI296341-FR and declined throughout fruit development and ripening. Expression of Cla006655 (*UGT*) was significantly higher in 97103 than that of PI296341-FR at 10 DAP and then decreased with fruit development in both accessions. Cla020180 (*SnRK2*) highly expressed in PI296341-FR exhibited a decreasing pattern with fruit development. The above results indicate that genes involved in ABA metabolism and signal transduction are differentially regulated at the transcriptional level between cultivated and wild watermelons.

### Genetic variation in the promoter region of ABA pathway genes could be associated with watermelon fruit evolution

**Fig 5:**
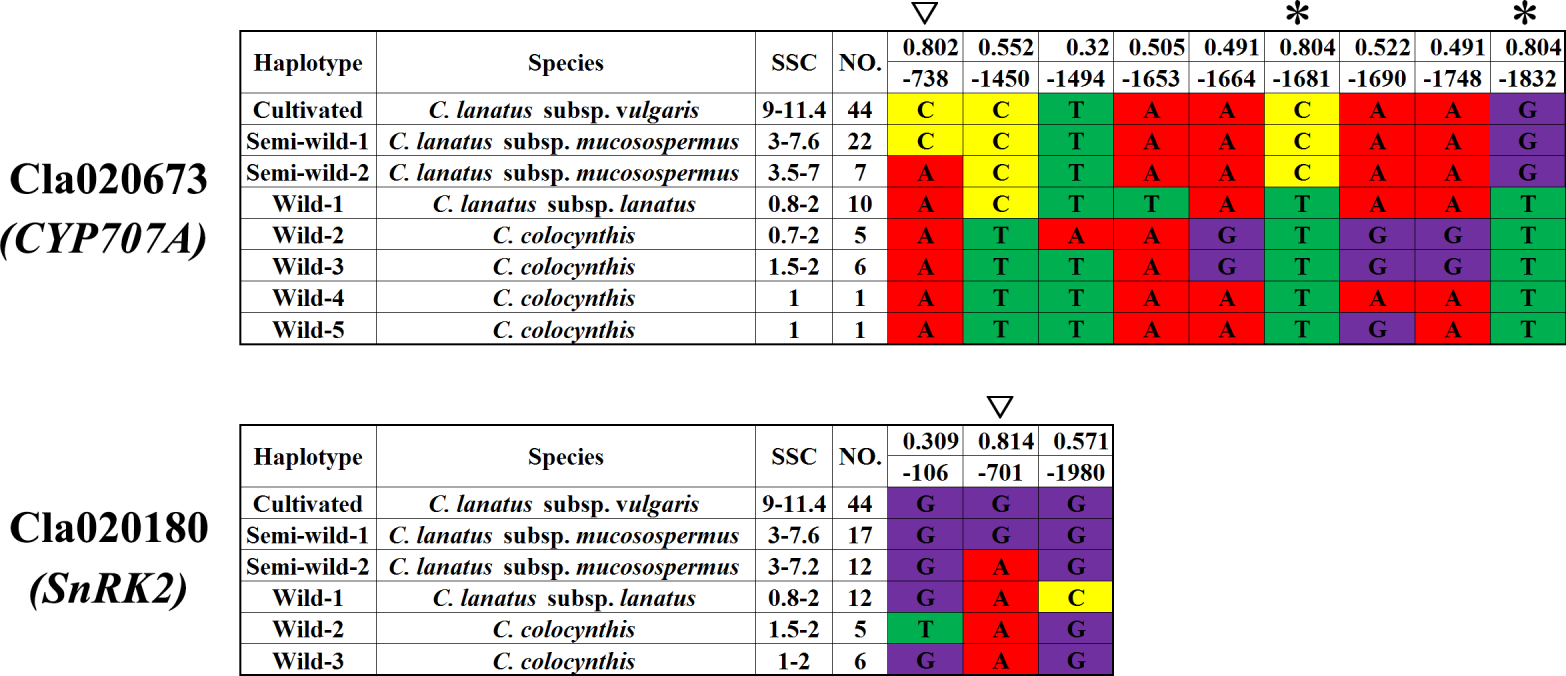
Genetic variations of Cla020673 (*CYP707A*) and Cla020180 (*SnRK2*) in the putative promoter regions of different watermelon accessions. The minus numbers represent the SNP position up the start codon and numbers above stand for the correlation of the corresponding SNP with SSC. The SNPs with asterisk and inverted triangle are the ones that have higher correlation values.

Our results indicated that five developmental-regulated ABA pathway genes including Cla005404 (*NCED*), Cla009779 (*NCED*), Cla020673 (*CYP707A*), Cla006655 (*UGT*) and Cla020180 (*SnRK2*) were differentially expressed in the center flesh of cultivated watermelon 97103 and wild watermelon PI296341-FR. To determine the reason underlying these differences in expression, we analyzed ~2 kb putative promoter sequences from 100 watermelon accessions, including 26 unsweet watermelon accessions of *C*. *colocynthis* and *C*. *lanatus* subsp. *lanatus*, 30 egusi type watermelons (also known as *C*. *lanatus* subsp. *mucosospermus*), and 44 sweet-desert cultivars (*C*. *lanatus* subsp. *vulgaris*) (Fig 5 and S3 Table). It seemed that these ABA metabolism and signal transduction genes were fixed during the evolution and domestication history of watermelon genome and fruit ripening. Another interesting result worth noting was that three SNPs (-738, C/A; -1681, C/T; -1832, G/T) in the promoter region of Cla020673 (*CYP707A*) and one single SNP (-701, G/A) in the promoter region of Cla020180 (*SnRK2*) explained most of the sugar content variation among the 100 germplasm accessions (r = 0.802, 0.804, 0.804, 0.814; Fig 5 and S3 Table).

## Discussion

### ABA is a key regulator in controlling watermelon fruit ripening

The evolution of watermelon fruits is a very interesting and complicated process, involving not only a shift in fruit size (from small to large) as occurred in fruits of most other plant species during evolution and domestication [29, 32], but also changes in fruit ripening characteristics from non-ripening (white and hard flesh, thick rind, bitterness) to ripening (red and crisp flesh, thin rind, sweetness) [27, 29]. To fully understand the process by which watermelon fruit ripening evolved, it is necessary to first define the mechanisms and processes responsible for inducing and regulating cultivated watermelon fruit ripening.

Watermelon is classified as a non-climacteric fruit [33, 34, 35]. Previous studies on the ripening of non-climacteric fruits, particularly those in the *Cucurbitaceae* family, such as cucumber and *Cucumis melo*, indicated that ABA was likely to involve in the regulation of watermelon fruit ripening [8, 36]. Several pieces of data collected throughout the course of our current study support the hypothesis that increases in levels of ABA in watermelon fruits play a critical role in fruit ripening.

Firstly, the ABA content in fruits of both 97103 and PI179878 was higher in center flesh and placenta than that in seeds at the ripening stage (Fig 1), from which it can be concluded that the ABA in the center flesh may not be transported from seeds, or at least the ABA from the seeds may not account for a big portion in the total ABA content. Evidences supporting this speculation could be found in studies on the source of ABA in apple and grape fruits at late developmental stages, where ABA were actively metabolized in the fruits [37, 38]. Moreover, an ABA peak was recorded to appear earlier in the seedless watermelon flesh during fruit ripening than that in the seeded fruit flesh [39], which further indicating that watermelon flesh could synthesize ABA by itself. Most importantly, in this study, genes encoding key enzymes catalyzing the ABA metabolism were also found highly expressed in the fleshy part of watermelon fruit, which also confirmed the speculation that ABA can be synthesized in watermelon flesh at the transcriptional level.

Additionally, dramatic increases in ABA levels were observed in all the tested fleshy tissues of both 97103 and PI179878 at the ripening stage (Fig 1A and 1D). Similar phenomena were not only recorded during the ripening of other non-climacteric fruits, such as cucumber, melon, citrus, grape, sweet cherry and strawberry [8, 6, 7, 36, 40, 41], but also observed in climacteric fruits, such as tomato, avocado, and mango [42, 43, 44]. This dramatic change of ABA level has been demonstrated to be an important signal either in triggering the onset of fruit ripening or in regulating the key metabolism pathways of fruit ripening, such as cell wall degradation and pigment biosynthesis [17, 45].

Finally, altering the levels of ABA present in watermelon fruit via exogenous chemical treatments significantly altered the ripening process. Based on previous studies, any significant shifts in ABA content in fruits at stages after mature green impacts the fruit ripening process in both non-climacteric (grape, strawberry and cucumber) [8, 11, 12] and climacteric fruits (tomato, mango and avocado) [17, 44, 46]. Our data support previously published studies, and application of exogenous ABA and NDGA treatments led to either the promotion or inhibition of watermelon fruit ripening, respectively. ABA and NDGA treatments altered fruit ripening through either speeding up or slowing down the process of coloring, the accumulation of soluble solids, and the decrease of firmness in the center flesh; all of which indicated that, the accumulation of ABA is directly involved in the regulation of watermelon fruit ripening (Fig 2B, 2C and 2D). Additionally, consistent with the results of our ABA/NDGA application studies, a highly positive correlation was also found between ABA content and SSC at the ripening stage in a population containing ancestral, wild, semi-wild and cultivated watermelon accessions (Fig 2A and 2C). Finally, the expression patterns of key genes involved in ABA metabolism and signal transduction were highly correlated with those of sugar-, firmness- and color-related genes (S4 Table and S11 Fig). These data indicate that ABA may be involved in regulating of watermelon fruit ripening at the transcriptional level. Similar co-expression between ABA pathway genes and genes involved in sugar accumulation, firmness and pigment metabolism pathways were also observed in tomato and strawberry [17, 45, 47], which strengthens our understanding that the regulation of ABA on the transcription of ripening related genes may widely exist in both climacteric and non-climacteric fruits. As a whole, our data strongly support a role for ABA in fruit development, and the hypothesis that increases in ABA levels are essential for fruit ripening.

Ethylene is another important fruit ripening regulator. It was reported that ethylene emission from flesh was higher than that from whole fruits, and the normal-ripening line 97103 had higher flesh ethylene content than that in non-ripening line PI296341-FR [48], which indicates that ethylene could potentially play a role in normal-ripening watermelon flesh. Based on previous studies in tomato, ABA and ethylene have a complicate interaction during fruit ripening, a higher ethylene content was recorded in the ABA content decreased transgenic fruits or the ABA deficient mutant fruits [17, 45, 49], moreover, both ethylene synthesis and signaling pathways were affected in the *SlNCED1* RNAi fruits [45]. Therefore, this kind of interaction may also exist in watermelon and further studies are needed in the future.

### ABA level may be fine-tuned by the coactions of multiple *NCEDs* and *CYP707As* genes at transcriptional level during watermelon fruit ripening

In the past decade, the transcriptional regulation of ABA levels during fruit ripening has been widely studied in several horticultural plant species, and genes encoding 9-*cis*-epoxycarotenoid dioxygenase (NCED) and ABA 8′‐hydroxylases (CYP707As) have been reported to play key roles in the regulation of ABA levels during fruit ripening process. In most of the studied species, one or two genes in the *NCED* and *CYP707A* gene families, are highly expressed and appear to play the dominant role in regulating ABA biosynthesis or degradation in these species. Examples of this include *SlNCED1* and *SlCYP707A2* in tomato [16], *FaNCED2* and *FaCYP707A1* in strawberry [41], *PacNCED1* and *PacCYP707A2* in sweet cherry [40], and *CsNCED1* and *CsCYP707A1* in cucumber [8]. Our data, however, indicated that all the *ClNCEDs* and *ClCYP707As* shown in Fig 3 were likely to function in the regulation of ABA level in the center flesh and placenta tissues of both cultivated 97103 and semi-wild PI179878 during watermelon fruit development and ripening. Specifically, in the center flesh and placenta tissues of cultivated accession 97103, expression levels of the *ClNCED* genes assayed exhibited a pattern similar to the ABA content in the same tissues. These data support a model where increased expression of ABA biosynthetic genes leads to increased ABA accumulation in watermelon fruits, ultimately triggering the onset of fruit ripening [50]. In terms of the *CYP707A* genes, levels of both Cla020673 and Cla016011 decreased during ripening, and may play important roles in keeping levels of ABA low during the early stages of fruit development. Interestingly, and in contrast to the other two *CYP707As*, expression levels of Cla005457 showed an increasing trend and may involve in the degradation of ABA at the later stages of fruit ripening, potentially to prevent over-accumulation of ABA at this stage. In the semi-wild PI179878 accession, Cla005404 (*NCED*) and Cla009779 (*NCED*) may play regulatory roles in the early and late development stages of center flesh, respectively. Cla009779, on the other hand, may be more important in placenta tissues. The *CYP707A* family genes Cla005457 and Cla016011 may play a regulatory role in center flesh, while Cla020673 and Cla016011 may be more important in the placenta (Fig 3).

On the other aspect, the ripening characteristic of the semi-wild watermelon PI179878 accession was different from that of cultivated watermelon accession 97103, which was reflected primarily in the nonuniform ripening of different parts of the fruit, in which the placenta was more mature than the center flesh (Fig 1F). The ABA levels in the fruits corresponded to these ripening characteristics, and were higher in placenta than in center flesh (Fig 1A and 1D), potentially as a result of the increased levels of Cla009779 expression in the placenta described above (Fig 3A and 3K). Our results indicate that, in both cultivated and semi-wild watermelons, ABA levels in watermelon fruits can be fine-tuned during development and ripening through the coordinated action of multiple *NCEDs* and *CYP707As* genes. A similar phenomenon has previously been reported in melon, in which it was discovered that ABA biosynthesis during fruit development and ripening may be controlled by two *CmNCEDs* simultaneously [36], suggesting that this fine-tune mechanism observed in watermelon was not unique in the *Cucurbitaceae*. An additional interesting phenomenon observed in this study was that in the mesocarp of both cultivated and semi-wild watermelon accessions, there was no increase in *NCED* expression corresponding to the highest ABA levels observed in these tissues at 34 DAP (Fig 3F and 3G), indicating that the ABA present in the mesocarp may not be entirely synthesized locally, but at least partially transported from other tissues.

### ABA may involve in the evolution of watermelon fruit quality trait and ripening

As our data clearly indicated that ABA played an essential role in regulating watermelon fruit ripening, it led us to think about the question of whether ABA was also involved in the evolution of watermelon fruit quality trait and ripening. SSC is one of the main quality traits that distinguishes the cultivated watermelon from wild and semi-wild accessions. The results disclosed the potential role of ABA in watermelon fruit quality trait and ripening evolution coming from the distribution of a population with varying degrees of evolved ripening (containing the wild, semi-wild and cultivated watermelon accessions) in the two-dimensional diagram by ABA and SSC, from which it can be observed that watermelon accessions, particularly wild and cultivated ones, can be clustered based on their evolutionary stage and ABA content was positively correlated with SSC (Fig 2A), suggesting that ABA may be involved in the regulation of SSC variation during the watermelon evolution and domestication. Furthermore, in the three detailly investigated watermelon accessions, it was observed that decreased levels of ABA in the fruit and reduced absolute *NCED* gene expression roughly correlated with a decreased level of fruit evolution. Another piece of evidence that could support the involvement of ABA in watermelon fruit evolution is that ABA content in watermelon seed, which has no obvious sign of evolution is not obviously different among 97103, PI179878 and PI296341-FR (Fig 1C). Our data therefore indicate that ABA may be involved in the evolution of fruit ripening, particularly the evolution of SSC. However, it is important to note that fruit ripening is a complex process, involving multiple pathways and phytohormones, and controlled by multiple genes [2]. Furthermore, the continuous distribution of the semi-wild watermelon accessions in the two-dimensional space defined by SSC and ABA content could also support the above hypothesis. Therefore, it implies that there should be multiple genes involved in the turning of non-ripening fruit into the ripening fruit during the evolution and domestication of watermelon. Therefore, further investigation of our RNAseq libraries might can reveal additional genes and pathways involved in watermelon fruit ripening. Additional support for the role of ABA in fruit ripening was provided by our analysis of SNPs present in cultivated, semi-wild and wild accessions. We identified three SNPs (-738, C/A; -1681, C/T; -1832, G/T) in the promoter region of the ABA catabolism gene Cla020673 (*CYP707A*) and one SNP (-701, G/A) in the promoter region of the ABA signal transduction gene Cla020180 (*SnRK2*), which could explain most of the SSC variation in the different watermelon accessions tested (Fig 5). This finding suggested that both ABA metabolism and signal transduction pathways may be involved in the ripening and quality trait evolution. The importance of ABA signaling pathways is highlighted by the fact that exogenous ABA treatment could not turn on or accelerate the ripening process of either the wild PI296431-FR or the semi-wild accession PI179878 [51]. In addition to phytohormone accumulation and signaling, there is evidence that ripening is also regulated by both DNA methylation and the action of specific transcription factors [2]. Because of this, in the future it will be important to expand investigations into the role of ABA in fruit ripening to cover the expression patterns of ABA-related transcription factors and the impact of DNA methylation on ABA synthesis and signaling in watermelon accessions at different stages of evolution.

## Conclusions

Our physiological assay, metabolite quantification, and gene expression data indicate that watermelon ripening is regulated by the accumulation of ABA. Additionally, our data indicate that ABA metabolism can be fine-tuned through regulation of ABA synthesis and catabolism genes encoding ClNCEDs and ClCYP707As. In our study, ABA content in watermelons was correlated well with both the SSC of these fruits and the state of evolution of the watermelon accession. Additionally, SNPs in the promoter region of Cla020673 (*CYP707A*) and Cla020180 (*SnRK2*) showed a high degree of correlation with SSC variation in different watermelon accessions, which suggested that ABA might be involved in watermelon fruit quality trait and ripening evolution. Our work answers some basic questions about the mechanisms of watermelon fruit ripening, and opens a new avenue to explore in the area of watermelon evolution. To comprehensively reveal the mechanism of watermelon fruit ripening regulation during evolution, systematic evolutionary analyses based on resequencing of more representative watermelon germplasm are needed. Future experiments should focus on investigating the role of ABA pathway genes involved in regulating watermelon fruit ripening and evolution.

## Supporting information

S1 Table Watermelon accessions used in determination of SSC and ABA content. (XLSX)

S2 Table Specific primers used in this study. (XLSX)

S3 Table Promoter sequences and SSC in different watermelon accessions. SNP variation in the Cla009779 (*NCED*), Cla005404 (*NCED*), Cla020673 (*CYP707A*), Cla006655 (*UGT*) and Cla020180 (*SnRK2*) promoter region and SSC in 100 watermelon germplasm accessions.
(XLSX)

S4 Table Gene co-expression analysis. The detailed co-expression information of ABA pathway genes with sugar-, firmness-, and color-related genes. Only PCC values higher than 0.9 are shown.
(XLSX)

S1 Fig Sequence alignment of the NCED family. All sequence data mentioned in this article can be found in the GenBank and accession numbers are as follows: AtNCED2, NP_193569.1; AtNCED3, NP_188062.1; AtNCED5, NP_174302.1; AtNCED6, NP_189064.1; AtNCED9, NP_177960.1; PaNCED1, AAK00632.1; PaNCED3, AAK00623.1; SlNCED1, NP_001234455.1; SlNCED2, XP_004244807.1.
(JPG)

S2 Fig Sequence alignment of the CYP707A family. All sequence data mentioned in this article can be found in the GenBank and accession numbers are as follows: AtCYP707A1, NP_567581.1; AtCYP707A2, NP_180473.1; AtCYP707A3, NP_851136.1; AtCYP707A4, NP_566628.1; PvCYP707A1, ABC86558.1; PvCYP707A2, ABC86559.1; PvCYP707A3, ABC86560.1; SlCYP707A1, NP_001234517.1; SlCYP707A2, XP_004244436.1.
(JPG)

S3 Fig Sequence alignment of the ZEP family. All sequence data mentioned in this article can be found in the GenBank and accession numbers are as follows: AtABA1, NP_851285.1; HP3, NP_001296233.1; NpABA2, Q40412.1; OsABA1, XP_015636352.1.
(TIF)

S4 Fig Sequence alignment of the AO family. All sequence data mentioned in this article can be found in the GenBank and accession numbers are as follows: AAO3, NP_180283.1; PsAO3, ABS32110.1; sitiens, XP_004228468.1.
(TIF)

S5 Fig Sequence alignment of the MOSU family. All sequence data mentioned in this article can be found in the GenBank and accession numbers are as follows: AtABA3, NP_564001.1; FLACCA, AAL71858.1.
(TIF)

S6 Fig Sequence alignment of the BG family. All sequence data mentioned in this article can be found in the GenBank and accession numbers are as follows: AtBG1, NP_175649.1; AtBG2, NP_180845.2; FaBG3, XP_004295227.1; SlBG2, XP_004244167.1; VvBG1, CBI27264.3.
(TIF)

S7 Fig Sequence alignment of the UGT family. All sequence data mentioned in this article can be found in the GenBank and accession numbers are as follows: UGT71B6, NP_188815.2; UGT71B7, NP_188816.1; UGT71B8, NP_188817.1; UGT71C5, NP_172204.1; UGT73B1, NP_567955.1; UGT73B3, NP_567953.1; UGT75B1, NP_563742.1; UGT75B2, NP_172044.1; UGT84B1, NP_179907.1; UGT84B2, NP_179906.1.
(TIF)

S8 Fig Sequence alignment of the PYL family. Residues made up the ligand-binding pocket are marked with black triangles. The cap and lock domains are noted. Functional residues and functional domains are based on the research of Melcher et al. (2009) and Santiago et al. (2009). All sequence data mentioned in this article can be found in the GenBank and accession numbers are as follows: AtPYL1, At5g46790; AtPYL2, At2g26040; AtPYL3, At1g73000; AtPYL4, At2g38310; AtPYL5, At5g05440; AtPYL6, At2g40330; AtPYL7, At4g01026; AtPYL8, At5g53160; AtPYL9, At1g01360; AtPYL10, At4g27920; AtPYL11, At5g45860; AtPYL12, At5g45870; AtPYL13, At4g18620; AtPYR1, At4g17870.
(TIF)

S9 Fig Sequence alignment of the PP2C family. Residues interacting with ABA, PYLs and Mn/Mg ions are marked with black triangles, asterisks and white triangles, respectively. Phosphatase sites are marked with black circles. Functional domains are based on the researches of Melcher et al. [52] and Santiago et al. [53]. All sequence data mentioned in this article can be found in the GenBank and accession numbers are as follows: ABI1, At4g26080; ABI2, At5g57050; HAB1, At1g72770; HAB2, At1g17550; AtPP2CA, At3g11410; AHG1, At5g51760; FaABI1, XP_011467233.1.
(TIF)

S10 Fig Sequence alignment of the subclass III SnRK2 family. Possible phosphatase sites are marked with black circles according to Umezawa et al. [54]. Functional residues and domains are noted according to Yoshida et al. [55]. All sequence data mentioned in this article can be found in the GenBank and accession numbers are as follows: AtSnRK2.2, At3g50500; AtSnRK2.3, At5g66880; AtSnRK2.6, At4g33950. (TIF)
(TIF)

S11 Fig Differentially expressed ABA pathway genes and the co-expression network. (A) Venn diagram of differentially expressed genes (DEG) in the center flesh of 97103 and PI296341-FR during watermelon fruit development and putative ABA metabolism and signal transduction genes, the five common genes are listed; (B) Co-expression network of ABA pathway, sugar, color and firmness related genes. Genes with a node degree ≥30 are highlighted, among which circles represent ABA pathway genes, diamonds represent sugar related genes, hexagon represents color related genes and triangle represent firmness related genes.
(TIF)
