# Supplementary figures and images for: Abscisic acid pathway involved in the regulation of watermelon fruit ripening and quality trait evolution
Source: PLoS One. 2017 Jun 29;12(6):e0179944. doi: 10.1371/journal.pone.0179944 (PMC5491074; doi:10.1371/journal.pone.0179944)

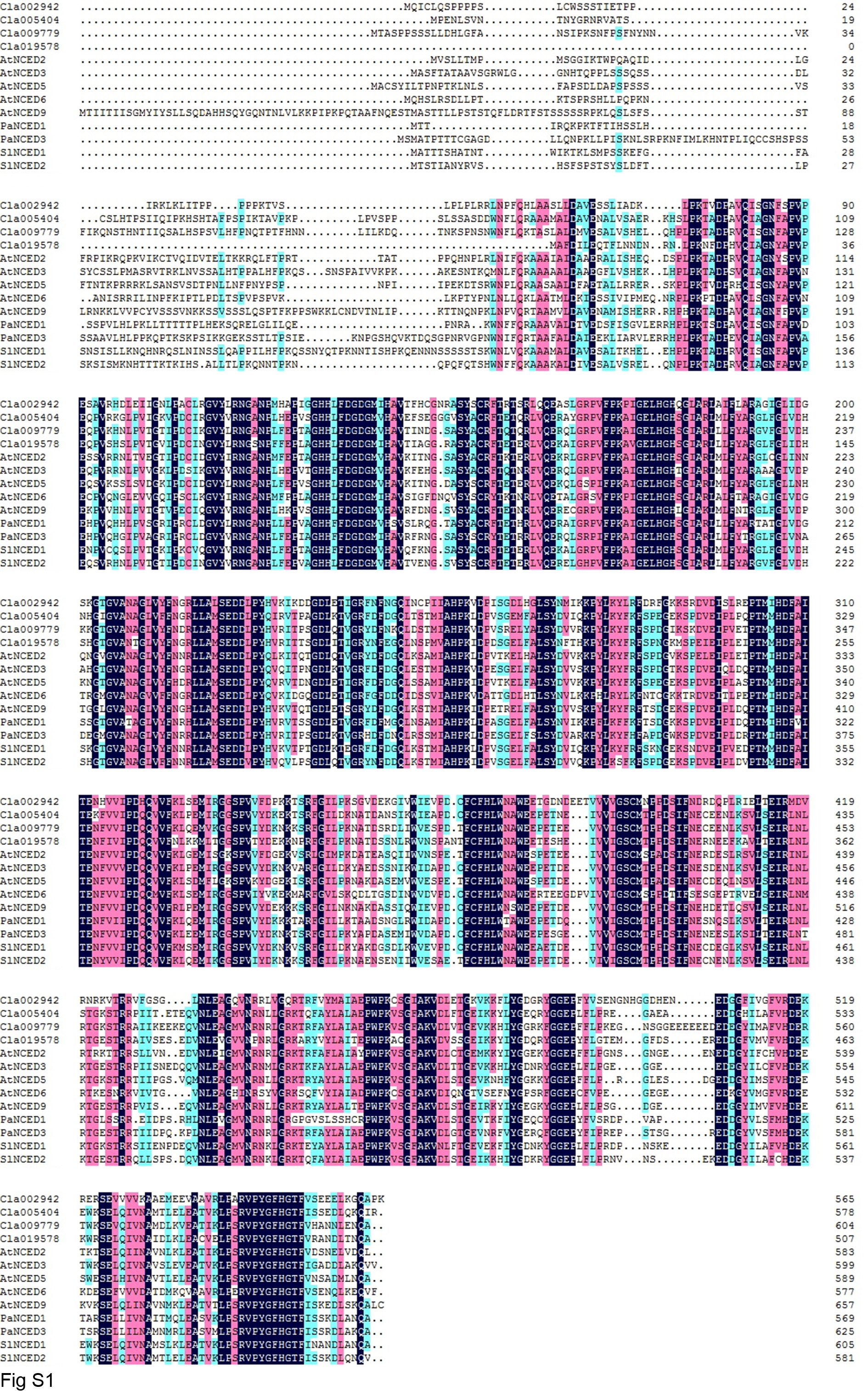

Supplement: S1 Fig — All sequence data mentioned in this article can be found in the GenBank and accession numbers are as follows: AtNCED2, NP_193569.1; AtNCED3, NP_188062.1; AtNCED5, NP_174302.1; AtNCED6, NP_189064.1; AtNCED9, NP_177960.1; PaNCED1, AAK00632.1; PaNCED3, AAK00623.1; SlNCED1, NP_001234455.1; SlNCED2, XP_004244807.1. (JPG) [file pone.0179944.s005.jpg]

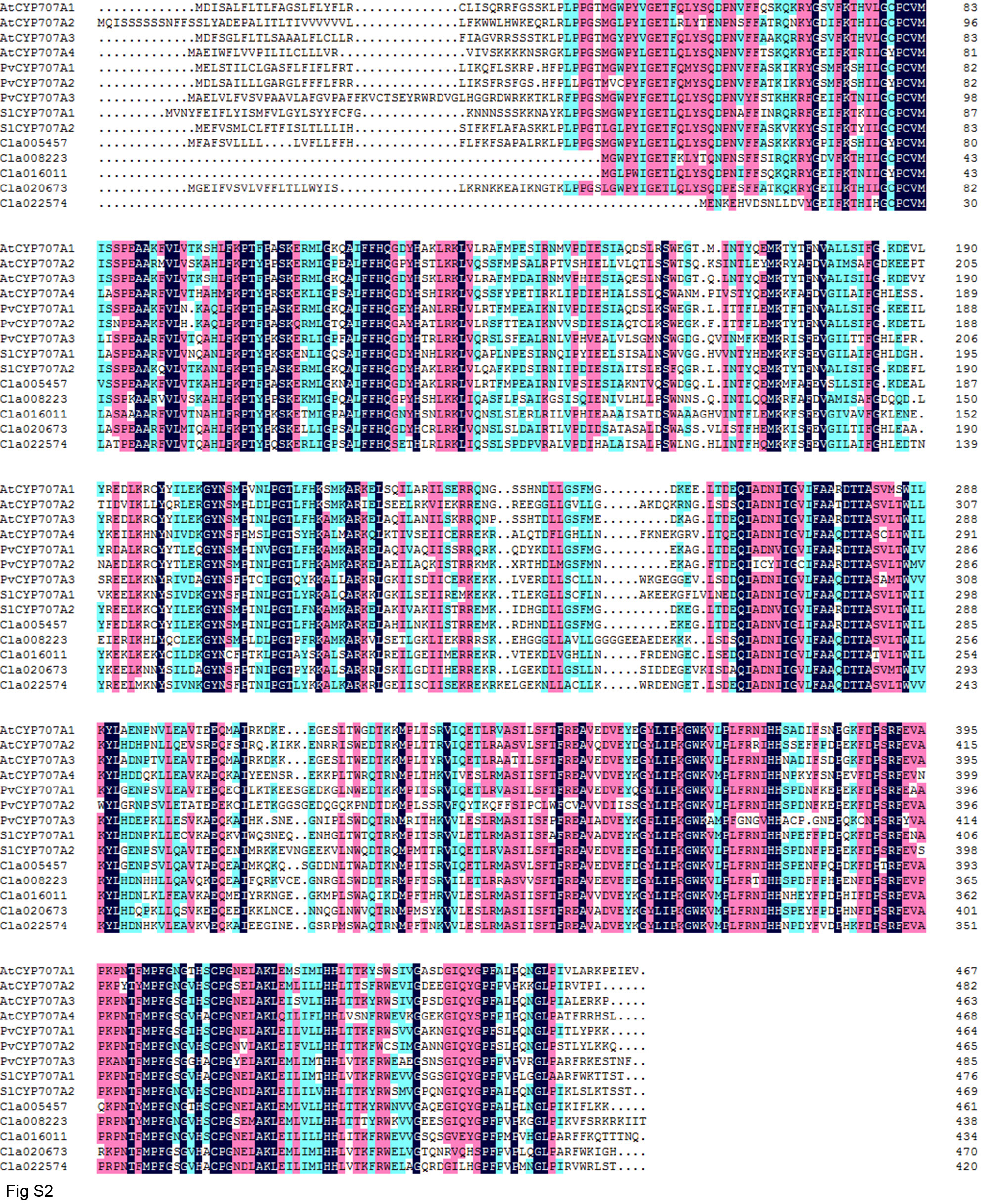

Supplement: S2 Fig — All sequence data mentioned in this article can be found in the GenBank and accession numbers are as follows: AtCYP707A1, NP_567581.1; AtCYP707A2, NP_180473.1; AtCYP707A3, NP_851136.1; AtCYP707A4, NP_566628.1; PvCYP707A1, ABC86558.1; PvCYP707A2, ABC86559.1; PvCYP707A3, ABC86560.1; SlCYP707A1, NP_001234517.1; SlCYP707A2, XP_004244436.1. (JPG) [file pone.0179944.s006.jpg]

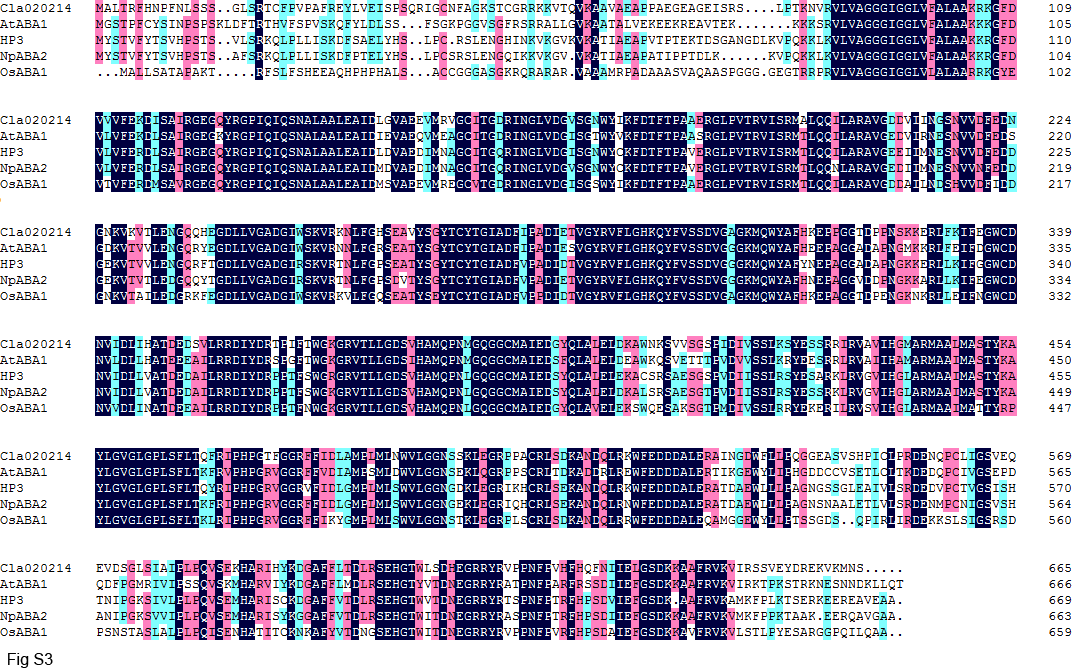

Supplement: S3 Fig — All sequence data mentioned in this article can be found in the GenBank and accession numbers are as follows: AtABA1, NP_851285.1; HP3, NP_001296233.1; NpABA2, Q40412.1; OsABA1, XP_015636352.1. (TIF) [file pone.0179944.s007.tif]

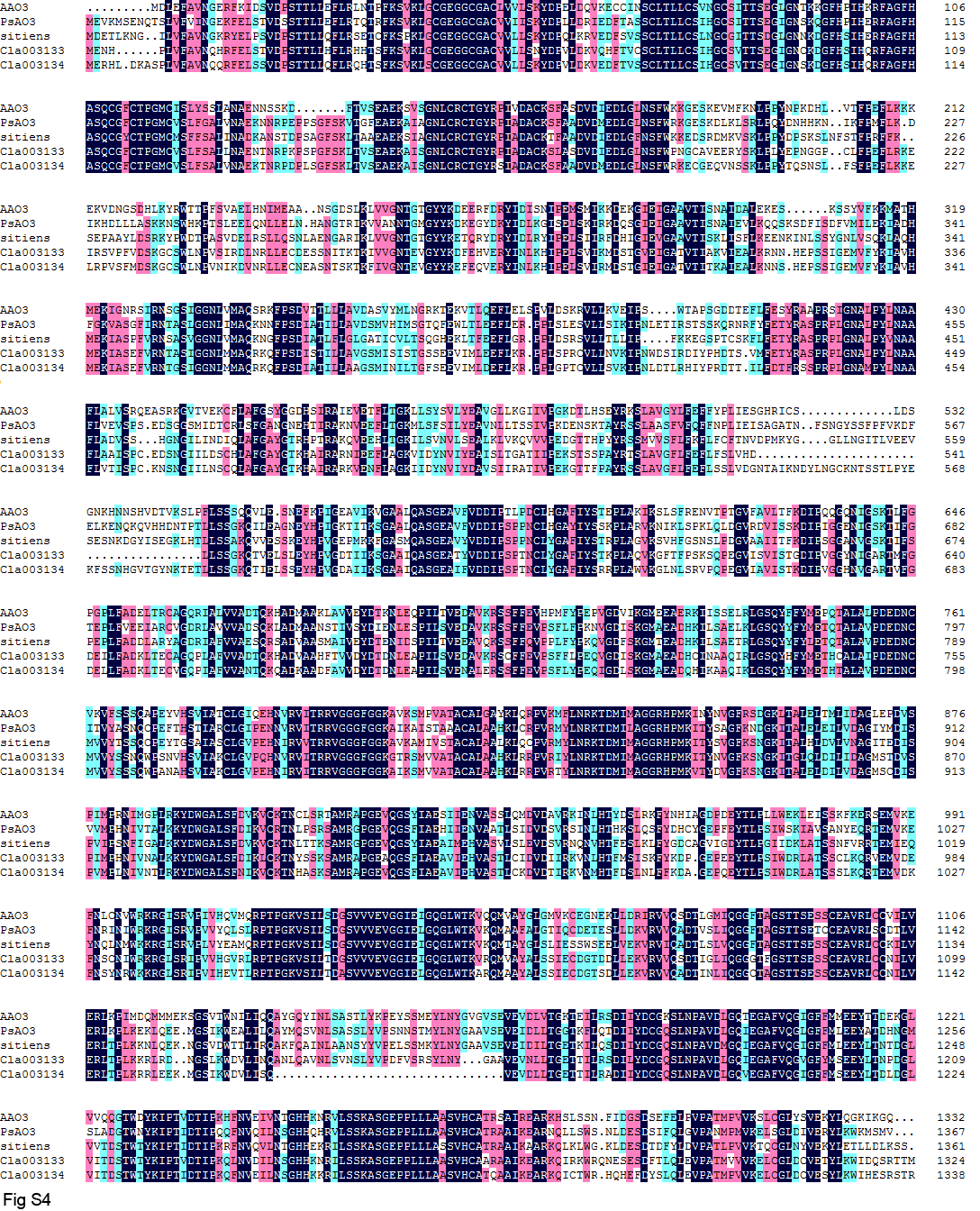

Supplement: S4 Fig — All sequence data mentioned in this article can be found in the GenBank and accession numbers are as follows: AAO3, NP_180283.1; PsAO3, ABS32110.1; sitiens, XP_004228468.1. (TIF) [file pone.0179944.s008.tif]

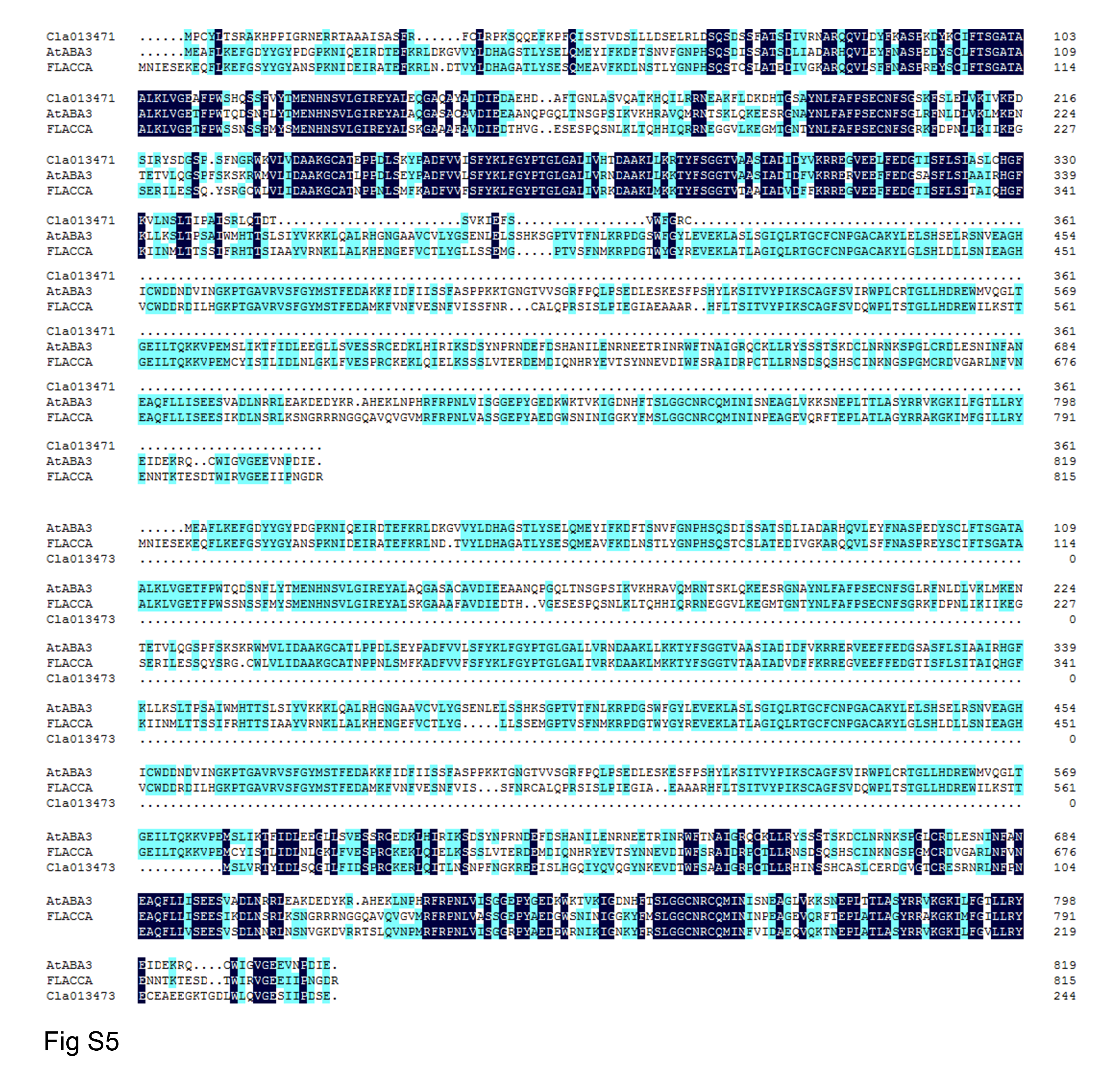

Supplement: S5 Fig — All sequence data mentioned in this article can be found in the GenBank and accession numbers are as follows: AtABA3, NP_564001.1; FLACCA, AAL71858.1. (TIF) [file pone.0179944.s009.tif]

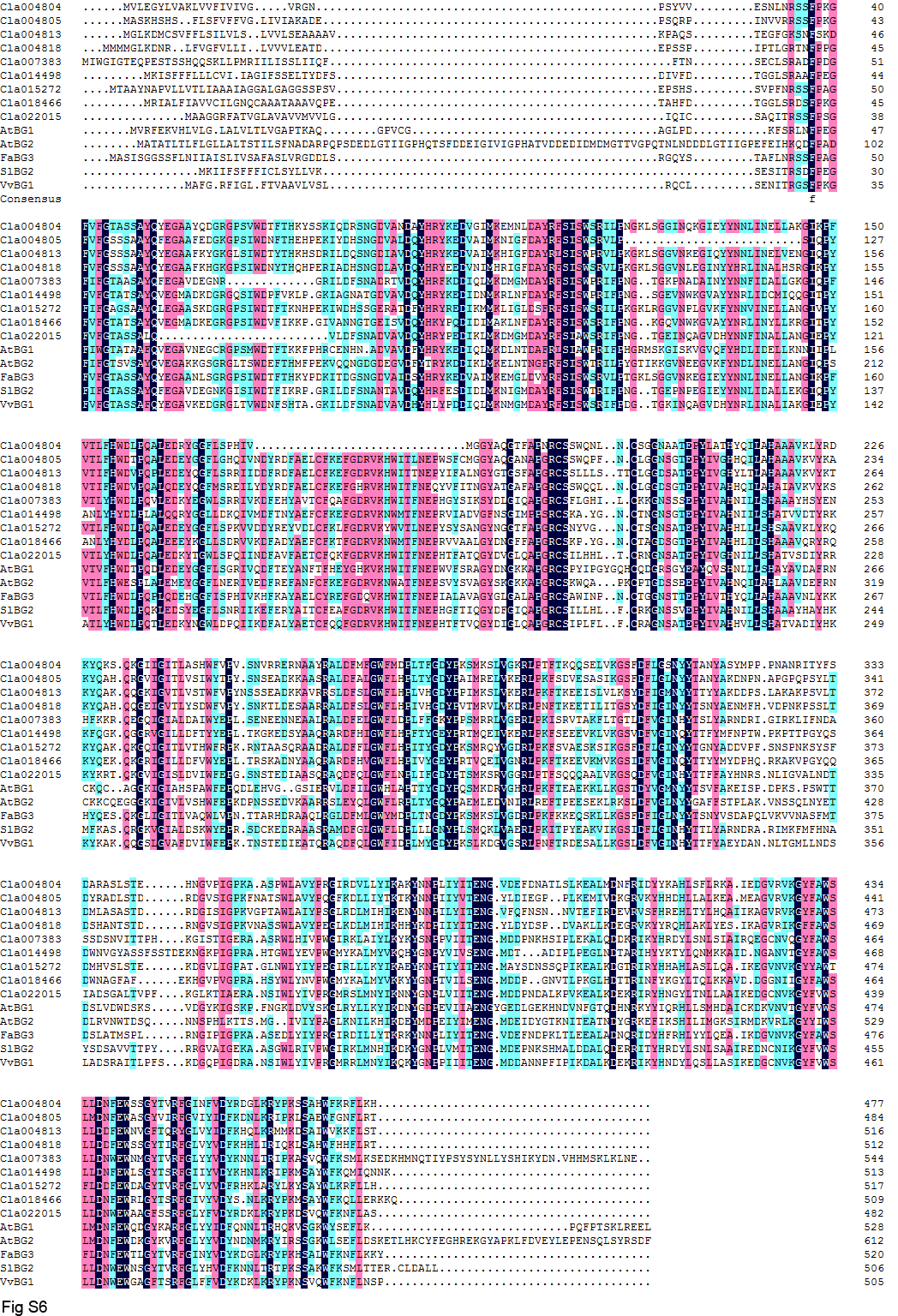

Supplement: S6 Fig — All sequence data mentioned in this article can be found in the GenBank and accession numbers are as follows: AtBG1, NP_175649.1; AtBG2, NP_180845.2; FaBG3, XP_004295227.1; SlBG2, XP_004244167.1; VvBG1, CBI27264.3. (TIF) [file pone.0179944.s010.tif]

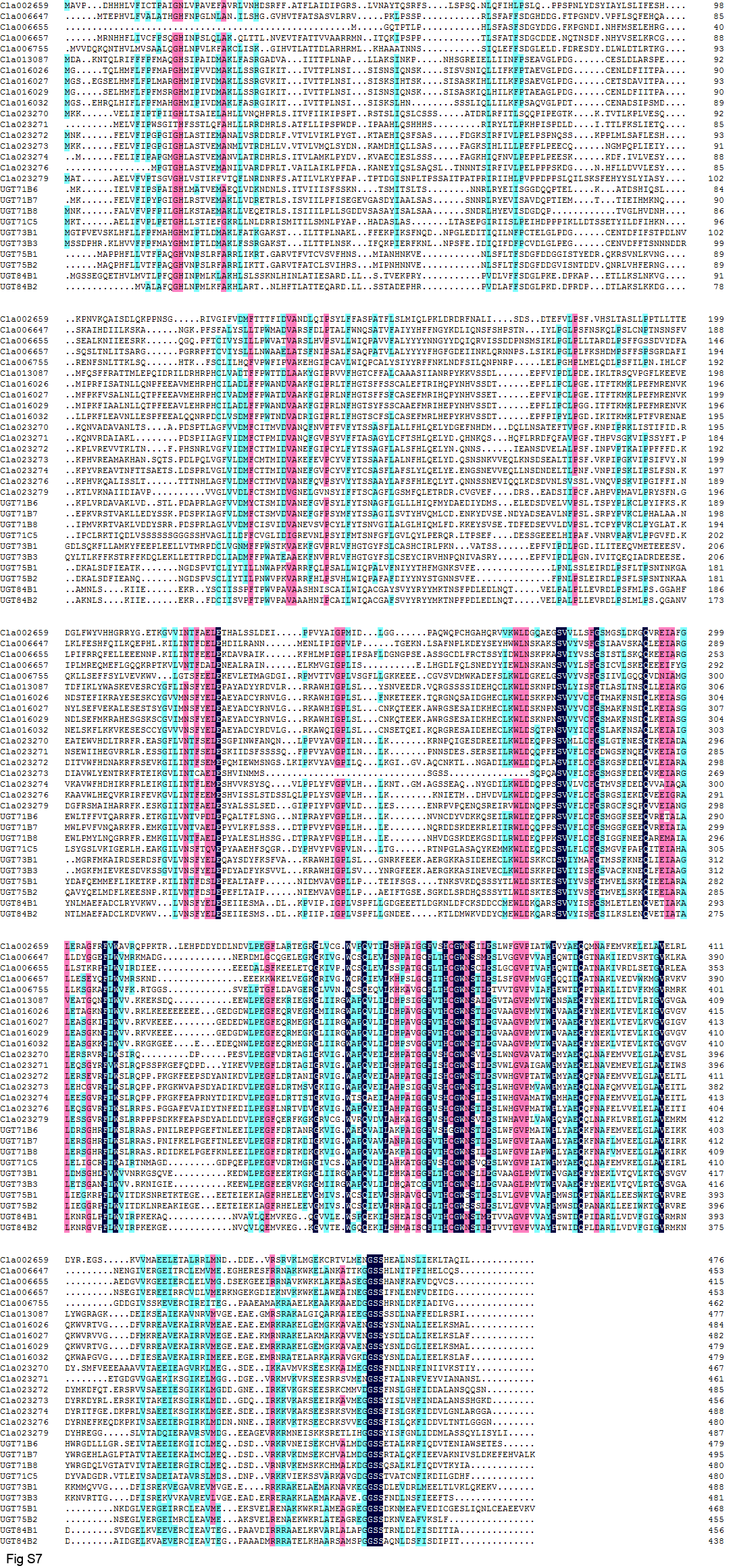

Supplement: S7 Fig — All sequence data mentioned in this article can be found in the GenBank and accession numbers are as follows: UGT71B6, NP_188815.2; UGT71B7, NP_188816.1; UGT71B8, NP_188817.1; UGT71C5, NP_172204.1; UGT73B1, NP_567955.1; UGT73B3, NP_567953.1; UGT75B1, NP_563742.1; UGT75B2, NP_172044.1; UGT84B1, NP_179907.1; UGT84B2, NP_179906.1. (TIF) [file pone.0179944.s011.tif]

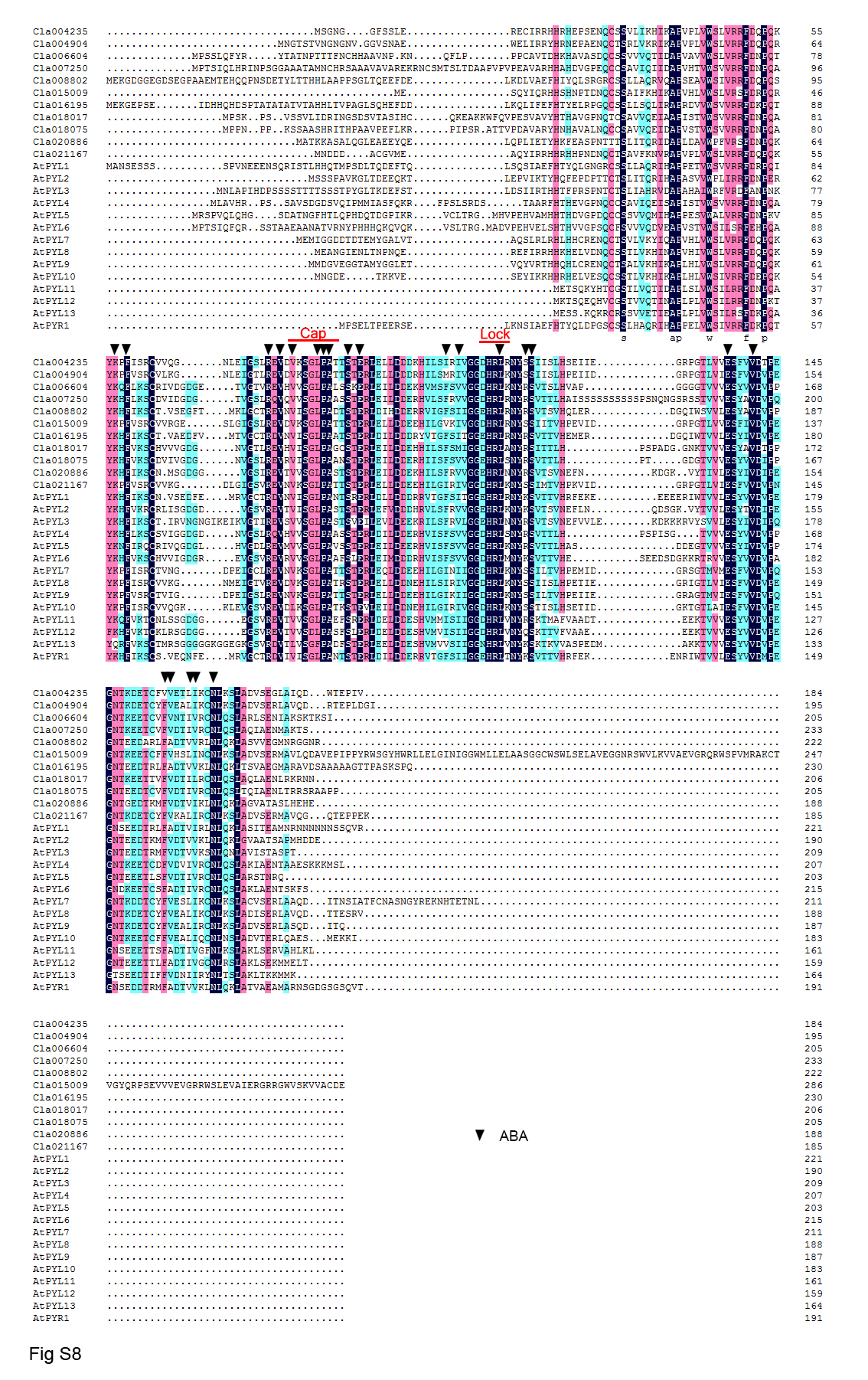

Supplement: S8 Fig — Residues made up the ligand-binding pocket are marked with black triangles. The cap and lock domains are noted. Functional residues and functional domains are based on the research of Melcher et al. (2009) and Santiago et al. (2009). All sequence data mentioned in this article can be found in the GenBank and accession numbers are as follows: AtPYL1, At5g46790; AtPYL2, At2g26040; AtPYL3, At1g73000; AtPYL4, At2g38310; AtPYL5, At5g05440; AtPYL6, At2g40330; AtPYL7, At4g01026; AtPYL8, At5g53160; AtPYL9, At1g01360; AtPYL10, At4g27920; AtPYL11, At5g45860; AtPYL12, At5g45870; AtPYL13, At4g18620; AtPYR1, At4g17870. (TIF) [file pone.0179944.s012.tif]

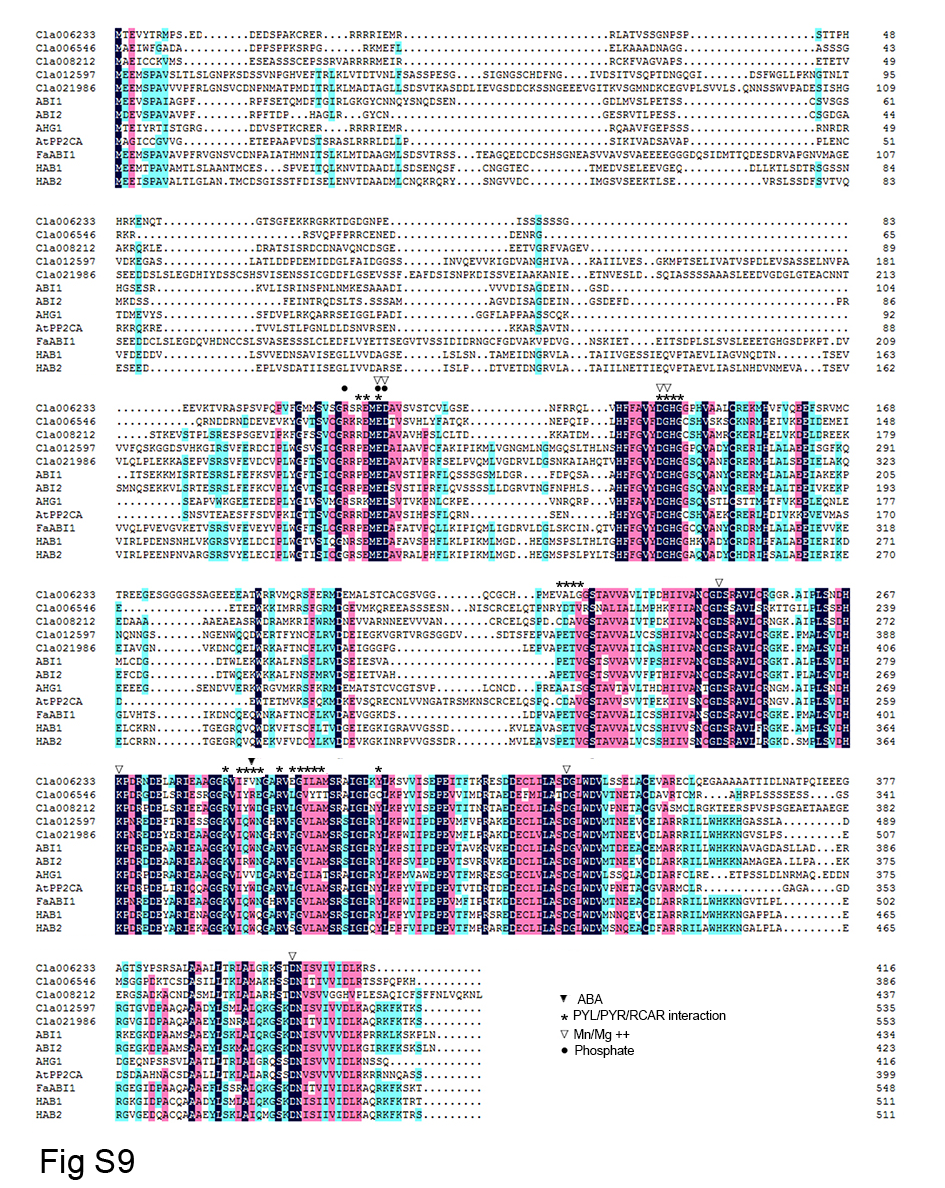

Supplement: S9 Fig — Residues interacting with ABA, PYLs and Mn/Mg ions are marked with black triangles, asterisks and white triangles, respectively. Phosphatase sites are marked with black circles. Functional domains are based on the researches of Melcher et al. [52] and Santiago et al. [53]. All sequence data mentioned in this article can be found in the GenBank and accession numbers are as follows: ABI1, At4g26080; ABI2, At5g57050; HAB1, At1g72770; HAB2, At1g17550; AtPP2CA, At3g11410; AHG1, At5g51760; FaABI1, XP_011467233.1. (TIF) [file pone.0179944.s013.tif]

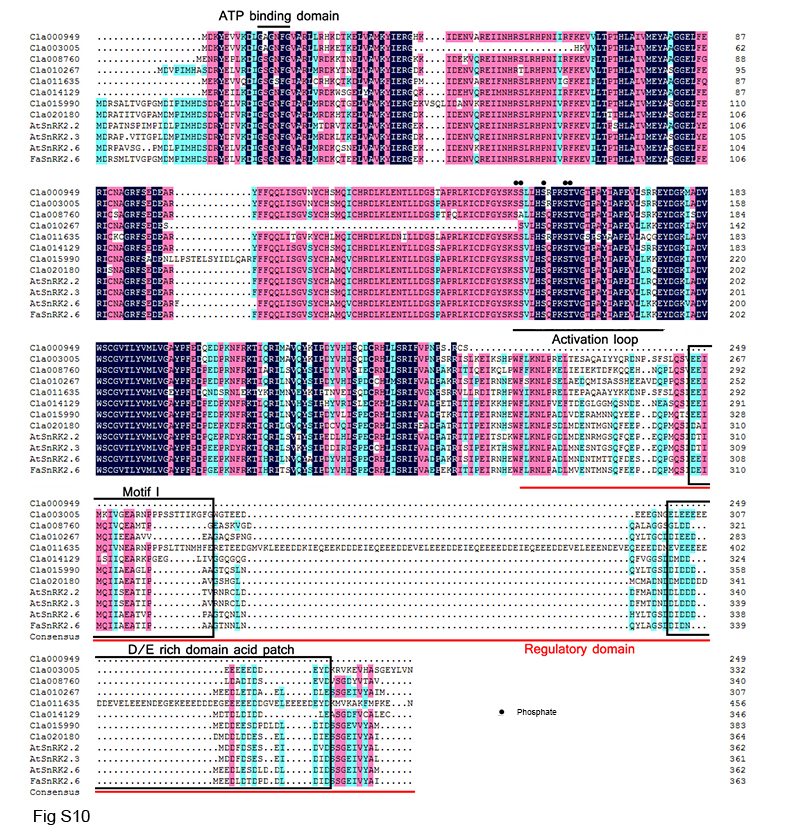

Supplement: S10 Fig — Possible phosphatase sites are marked with black circles according to Umezawa et al. [54]. Functional residues and domains are noted according to Yoshida et al. [55]. All sequence data mentioned in this article can be found in the GenBank and accession numbers are as follows: AtSnRK2.2, At3g50500; AtSnRK2.3, At5g66880; AtSnRK2.6, At4g33950. (TIF) (TIF) [file pone.0179944.s014.tif]

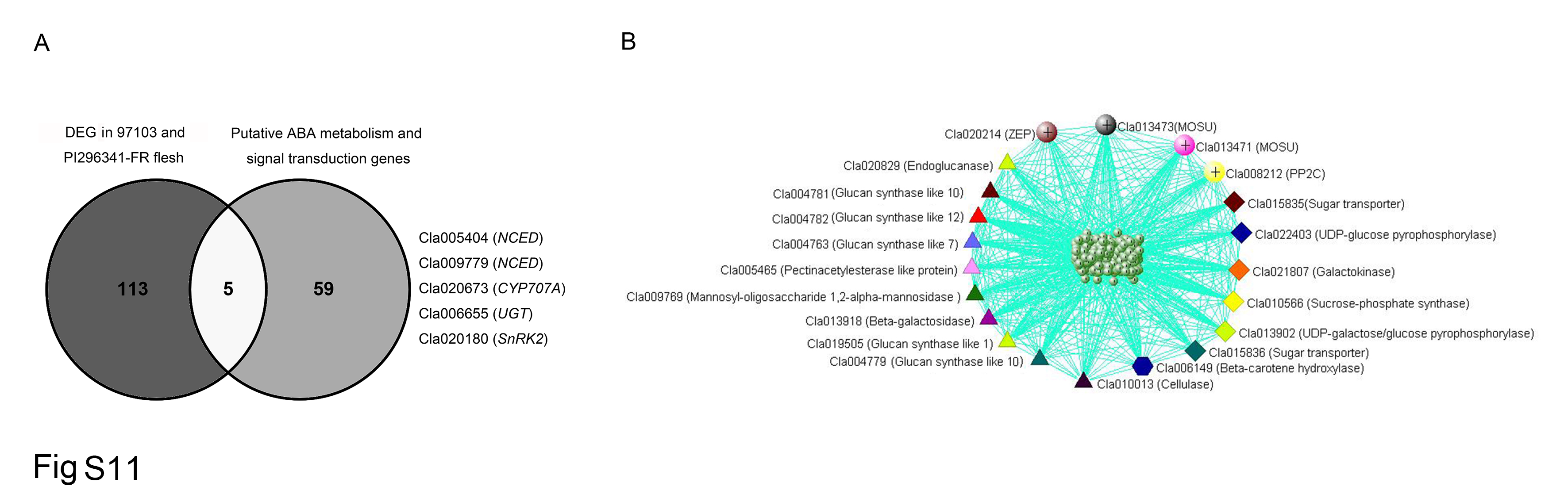

Supplement: S11 Fig — (A) Venn diagram of differentially expressed genes (DEG) in the center flesh of 97103 and PI296341-FR during watermelon fruit development and putative ABA metabolism and signal transduction genes, the five common genes are listed; (B) Co-expression network of ABA pathway, sugar, color and firmness related genes. Genes with a node degree ≥30 are highlighted, among which circles represent ABA pathway genes, diamonds represent sugar related genes, hexagon represents color related genes and triangle represent firmness related genes. (TIF) [file pone.0179944.s015.tif]
